# Mitigating Financial Burden of Tuberculosis through Active Case Finding Targeting Household and Neighbourhood Contacts in Cambodia

**DOI:** 10.1371/journal.pone.0162796

**Published:** 2016-09-09

**Authors:** Fukushi Morishita, Rajendra-Prasad Yadav, Mao Tan Eang, Saly Saint, Nobuyuki Nishikiori

**Affiliations:** 1 World Health Organization Regional Office for the Western Pacific, Manila, Philippines; 2 World Health Organization Representative Office in the Philippines, Manila, Philippines; 3 National Center for Tuberculosis and Leprosy Control, Ministry of Health, Phnom Penh, Cambodia; Mahidol-Oxford Tropical Medicine Research Unit, THAILAND

## Abstract

**Background:**

Despite free TB services available in public health facilities, TB patients often face severe financial burden due to TB. WHO set a new global target that no TB-affected families experience catastrophic costs due to TB. To monitor the progress and strategize the optimal approach to achieve the target, there is a great need to assess baseline cost data, explore potential proxy indicators for catastrophic costs, and understand what intervention mitigates financial burden. In Cambodia, nationwide active case finding (ACF) targeting household and neighbourhood contacts was implemented alongside routine passive case finding (PCF). We analyzed household cost data from ACF and PCF to determine the financial benefit of ACF, update the baseline cost data, and explore whether any dissaving patterns can be a proxy for catastrophic costs in Cambodia.

**Methods:**

In this cross-sectional comparative study, structured interviews were carried out with 108 ACF patients and 100 PCF patients. Direct and indirect costs, costs before and during treatment, costs as percentage of annual household income and dissaving patterns were compared between the two groups.

**Results:**

The median total costs were lower by 17% in ACF than in PCF ($240.7 [IQR 65.5–594.6] vs $290.5 [IQR 113.6–813.4], p = 0.104). The median costs before treatment were significantly lower in ACF than in PCF ($5.1 [IQR 1.5–25.8] vs $22.4 [IQR 4.4–70.8], p<0.001). Indirect costs constituted the largest portion of total costs (72.3% in ACF and 61.5% in PCF). Total costs were equivalent to 11.3% and 18.6% of annual household income in ACF and PCF, respectively. ACF patients were less likely to dissave to afford TB-related expenses. Costs as percentage of annual household income were significantly associated with an occurrence of selling property (p = 0.02 for ACF, p = 0.005 for PCF).

**Conclusions:**

TB-affected households face severe financial hardship in Cambodia. ACF has the great potential to mitigate the costs incurred particularly before treatment. Social protection schemes that can replace lost income are critically needed to compensate for the most devastating costs in TB. An occurrence of selling household property can be a useful proxy for catastrophic cost in Cambodia.

## Introduction

Tuberculosis (TB) is predominantly a disease of the poor [1]. For the past decades, the global TB community has strived to address the needs of poor and marginalized population through promoting pro-poor strategies in TB control programmes [1]. The international standard has been established that basic TB diagnostic and treatment services are provided free of charge [2, 3]. Nevertheless, TB patients often face severe financial burden by spending considerable amount of out-of-pocket (OOP) expenses before and during treatment [3, 4]. They are often trapped in a vicious cycle of repeated visits at the same healthcare level [5] or complex care-seeking pathways at multiple healthcare providers including private facilities and traditional healers unlinked to the national TB programme (NTP) [6], escalating their OOP expenditures. Free TB services help reduce direct medical cost borne by the patient, however in reality there are other hidden costs such as direct non-medical costs (i.e. costs for food, transportation and accommodation) and indirect costs (i.e. lost income and reduced productivity) [7, 8]. A recent systematic review that involved 49 studies from 32 low- and middle-income countries (mostly African and Asian countries with some Latin American countries) revealed that indirect cost accounted for 60% of the total cost faced by patients across 25 surveys that provided the disaggregated data, constituting the largest financial risk for patients [9]. The total direct and indirect cost can be significant, being equivalent to 39% of reported household income [9]. The financial barriers to accessing TB services, often coupled with geographical and health system barriers, contribute to delayed diagnosis, leading to more advanced disease and continued transmission, and resulting in poor health outcomes and further aggravating poverty for the patient and affected household [7–9].

The WHO End TB Strategy highlighted the need for accelerated progress toward universal access and social protection [10]. The Strategy aims to achieve that no TB affected families experience catastrophic costs due to TB by 2020 [11]. To monitor the progress toward this target, WHO has been exploring the definition of TB-specific “catastrophic costs” taking into account the hidden costs [12]. This is in contrast to the indicator of “catastrophic health care expenditure” which WHO defined as direct health expenditures (not including indirect cost) of >40% of annual discretionary income [3, 12]. The two options being considered are (1) the percentage of TB-affected households facing a total cost that is above a certain percentage of annual household income, and (2) the percentage of TB-affected households experiencing “dissaving” (such as taking a loan or selling assets) to cope with TB-related expenses as a proxy for catastrophic costs [12]. Although several studies are available that documented direct and indirect patient cost as a percentage of household income [9, 13], only three studies published recent data after 2010 [13–15]. A study conducted in Peru suggested a threshold of total expenses ≥ 20% of annual household income as it was associated with poor clinical TB outcomes [14]. Since the effort to explore the TB-specific “catastrophic costs” is relatively new, few studies provided a comprehensive set of data that allows to examine changes in the proportion of patients facing catastrophic costs with different thresholds using empirical data. For the second option to be chosen, the correlation between coping strategies and high total cost relative to income needs to be assessed [9]. So far, only one study was available that examined this association. A significant positive association was found between the occurrence of dissaving and total costs incurred in Tanzania and India [16]. In Bangladesh, an increase in dissaving of $10 US dollar (USD) was significantly associated with an increase in total cots of $7 USD among low-income patients [16]. More evidence needs to be accumulated from different countries and contexts.

To mitigate financial hardship of TB patients and overcome access barriers, various interventions have been implemented in many parts of the world [3, 4, 17]. One approach is to provide direct or indirect economic support for patients or affected-households through the provision of, for example, nutrition package, food package, transport allowance/vouchers/reimbursement, occupational training, and income generating fund [4]. Active case finding (ACF) for TB, if implemented deliberately with strategic selection of target population and diagnostic algorithms, has a potential to improve access and detect prevalent cases earlier [18–20]. Early case finding brought about by ACF may further help prevent unnecessary OOP expenditure and income losses, and thereby reduce associated costs for patients and affected household. However the quantitative evidence to demonstrate these benefits is limited, with no studies so far comparing patient costs between actively- and passively-detected patients.

Cambodia is a low-income country and one of 22 countries with a high burden of TB [21]. The Cambodian National Centre for Tuberculosis and Leprosy Control (CENAT) has progressively intensified case finding activities for TB to ensure equitable access to quality TB services [22]. For the last several years, CENAT has adopted many systematic case-finding approaches, including ACF, to bring TB services closer to hard-to-reach populations [23, 24]. Since 2005, CENAT has conducted ACF targeting household and neighbourhood contacts in poor communities alongside routine passive case finding (PCF), a symptom-driven facility-based case finding approach. The results from the national TB prevalence surveys in 2002 and 2011 showed a slow decline of smear-positive TB prevalence rate for asymptomatic cases, and highlighted the limitation of DOTS strategy which has focused on passive detection by smear microscopy among symptomatic individuals [25]. Then, after 2012, CENAT upgraded the ACF strategy by introducing Xpert MTB/RIF (Cepheid, Sunnyvale, CA, USA) to enable better diagnostic capacities especially for asymptomatic and sputum smear-negative patients with the funding from TB REACH. The previous study showed this Cambodia’s ACF among contacts tended to find more patients from an older age group and more patients who were smear-negative or had lower smear grades, as compared to PCF, showing an indication of early case finding [26]. Furthermore, the ACF increased case detection beyond what is reported in PCF [27], and was found to be highly cost-effective from a provider perspective [28].

To date, there was only one study that surveyed TB-affected household costs in Cambodia [29]. The survey took place in 2008/2009 and showed that the average total household cost was US$476.8 per TB episode, ranging from US$395 to $1900, depending on the modality of care [29]. However, the study did not provide costs as percentage of household income nor information about dissaving. Furthermore these cost data need to be updated in light of the WHO End TB Strategy to provide a baseline indicator of progress towards eliminating financial hardship due to TB.

Against this background, we aim to examine whether or to what extent the Cambodia’s ACF among household and neighbourhood contacts reduces financial burden of TB-affected household by comparing costs due to TB between actively- and passively-detected patients. The study further aims to examine the association of catastrophic cost with different dissaving patterns using different thresholds as well as to provide a baseline indicator to monitor and evaluate the progress towards eliminating financial hardship due to TB in the context of Cambodia.

## Methods

### Programmatic information

The intervention was conducted in socio-economically disadvantaged and underserved areas with a relatively high burden of TB. In the selected health centres, community volunteers and health workers conducted house-to-house visits of all smear-positive TB patients who had been registered for treatment during the preceding two years. All of their household contacts regardless of TB symptoms and symptomatic neighbourhood contacts with cough, fever, weight loss, and/or night sweats of more than two weeks were invited to the prescheduled ACF session on a specific date in their nearest health centres. Neighbourhood contacts were included in screening efforts as they are likely exposed to infectious index cases through close community interaction that is typical in rural areas. Two weeks prior to ACF session dates, CENAT-ACF team visited intervention sites to train existing health facility staff and selected community volunteers on the project initiative including screening, diagnosis and care procedures. During the two-week preparation period, the trained staff conducted house-to-house visits for pre-screening and inviting eligible participants to ACF. On the day of ACF session, all participants were re-screened for TB symptoms and a history of contact at ACF sites by clinicians of the CENAT team to verify the eligibility of the participants. They then underwent chest X-ray (CXR) examinations. CXR films were developed immediately and evaluated by a radiologist of the team by classifying either normal or abnormal. Abnormal CXR findings were further classified as active TB, suspected TB, healed TB, or other abnormalities to facilitate clinical diagnosis. Individuals who had abnormal CXR findings and/or TB symptoms were asked to provide a sputum specimen for Xpert MTB/RIF testing. Those with MTB-positive results were reported as bacteriologically-confirmed TB. Diagnosis of bacteriologically-negative TB and extra-pulmonary TB was made onsite by the clinicians in the CENAT-ACF team based on all available evidence, in principle, on the same day of the ACF session. Treatment of the detected patients was managed by routine health services.

### Study design and sampling

This is a cross-sectional comparative study involving a questionnaire survey that explores costs associated with TB diagnosis and treatment among actively- and passively-detected TB patients. The intervention group consisted of patients diagnosed through ACF sessions organized at health centres. The control group was taken from patients who were diagnosed and registered in the same health centres within four months prior to the respective ACF sessions. All participants were new pulmonary TB patients with treatment outcome of either “completed” or “cured”. For the simplicity of interpretation of results, patients with unfavourable treatment outcomes and retreatment and extra-pulmonary patients were not included in the study.

We employed a combination of purposive sampling (for district and health centre selection) and systematic sampling (for patient selection). Of 30 operational districts (ODs) with intervention in 2012 and 2013, four ODs (Oudong, Angkor Chey, Stoung and Sothnikum) were selected based on the implementation timing (ODs with ACF patients who had most recently completed treatment at the time of data collection) and geographical distribution (ODs that were near and far from the capital to allow geographical diversity). Within these ODs, we further targeted health centres with relatively high TB case notifications both in ACF and PCF to ensure the adequate number of eligible patients. Then we systematically approached the eligible participants in order from the top of the eligible subject list in each health centre. The difference in the sample size between ACF and PCF in each health centre was set at no more than five patients to avoid a biased representation of the two groups from each health centre. We used a prevalence of catastrophic costs as the outcome variable to guide sample size estimation with the formula described by Pocock [30]. Assuming that 20% in the control group and 5% in the intervention group had faced catastrophic costs due to TB, 194 patients (97 patients from each group) were required to have a 90% chance of detecting a difference in the two groups at the 5% significance level. As a result, we visited 25 health centres until we reached the sample size. Eligible participants were contacted by the health centre staff and community volunteers and invited to the pre-scheduled interview. Data collection took place between October and December 2014. A total of 108 ACF patients and 100 PCF patients were recruited for the study.

The interviews were conducted by three local research assistants either at the health centre or participants’ house in a private manner. The tool to estimate patients’ costs [7], a structured questionnaire, was adapted to the local setting, translated into Khmer, pre-tested with adjustment as needed, and administered during each interview. If a patient was under 18 years of age, their guardian was asked to answer the questions with or without participation of the patient. All participants or their guardians provided their written consent before commencement of interview. The ethical clearance was obtained from the Cambodian National Ethics Committee for Health Research before study commencement.

### Quantitative Data and Statistical Analysis

Demographic and clinical information were sourced from project database, TB registers, laboratory registers, and individual treatment card. The questionnaire included various questions on health-seeking behaviour, costs associated with TB diagnosis and treatment, and socio-economic information including household income and spending. We collected and calculated a wide range of cost data including direct medical cost, direct non-medical cost, indirect cost, reimbursement and coping costs. Direct medical cost included OOP expenses for facility administration/consultation, laboratory test, X-ray, drug, and hospitalization. Direct non-medical cost included OOP expenses for food, transportation, guardian and caregiver (food, transportation and accommodation for an escort), accommodation, supplemental foods given to patients during treatment, and interest for borrowed money. Indirect cost included patient’s lost income, guardian/caregiver’s lost income and value lost due to sold property. We also collected insurance reimbursement. Many of these costs were, where appropriate, collected separately for two different time periods, “before treatment initiation” and “during 6-months treatment period”.

To estimate direct costs before treatment, we obtained the data of actual OOP expenses for each different health-seeking visit related to the single episode of TB before treatment. To estimate direct costs during treatment, we obtained the data on the average costs per visit for the different items and they were multiplied by the number of visits to health facilities during treatment. Our questionnaire captured costs for three types of visits including daily DOT, picking-up drugs, and follow-up examinations. A visit with multiple purposes was counted as one visit.

For all health-seeking visits of all participants before treatment, we obtained the total health-seeking time in minutes including time spent for travel, waiting, consultation and hospitalization. For patients who had any income before TB illness, the total health-seeking time per patient was then multiplied by their income per minute before TB illness to estimate lost income due to health-seeking, assuming that a patient worked for 8 hours per day for 5 days per week. For ACF patients, health-seeking time during ACF session was included in the total health-seeking time. For 69 participants (40 ACF and 29 PCF patients), only travel time was available and other components of their health-seeking time were extrapolated using the data from the rest of the samples by types of health facilities visited. For participants who reported sick leave before treatment, we estimated lost income due to sick leave using their income per day before TB illness multiplied by the duration of sick leave in days before treatment.

Lost income during treatment was calculated for patients experiencing a change in the average weekly household income due to TB, using the reduced weekly income multiplied by patient’s actual treatment period in weeks. If patients who engaged in housework stopped their work due to TB and their labour force was replaced by someone else, we estimated cost of reduced household activity of patient using self-reported estimated value of the work per day multiplied by the duration of treatment.

If the patient was accompanied by a guardian on health facility visits during treatment, their lost income was estimated using the reported income per day multiplied by the number of accompanied visits. If caregivers of the patient quitted their jobs to specifically take care of the patient, their lost income due to caregiving was estimated using the reported lost income per week multiplied by duration of caregiving during treatment. Lost income of guardians and caregivers were combined and reported together. Value lost due to sold property was not an actual selling price but defined as the difference between an actual selling price and self-estimated market value of a sold property that was also asked in the interview.

To examine the changes in the proportion of catastrophic costs, costs as percentage of reported annual household income were calculated. The proportion of patients who spent total expenses ≥10%, ≥20%, ≥30% and ≥40% of annual household income were compared between ACF and PCF. The prevalence of dissaving was further explored by stratifying patients into four cost-income bands that were <10%, 10–20%, 20–30% and >30%.

For categorical data, distribution and frequency were presented, and a Pearson’s chi-square test was used to examine associations. For numerical data, we presented median, mean, inter-quartile range (IQR), standard deviation (SD) and range in tables. For most of the cost data that were skewed, the median was mainly reported in the text while the mean was also used when describing the proportion of sub-categorized costs among total costs as well as describing the cost data with an extremely skewed distribution where median and IQRs were all zero both in ACF and PCF. The Welch T-test was used for age comparison. A two-sample Wilcoxon rank-sum test was applied to examine the difference in various costs and costs as percentage of annual household income between ACF and PCF. A Fisher’s exact test was employed to assess the association between costs as percentage of annual household income and the prevalence of different dissaving patterns. Statistical significance is defined as p<0.05. All statistical analyses were performed using the statistical software package R 3.2.1 (CRAN: Comprehensive R Archive Network at https://cran.r-project.org/). All cost data were obtained in local currency (Cambodian Riel) and then were converted into USD for analysis at the rate of 4000 Riel per dollar.

## Results

### Participant characteristics (Table 1)

A total of 108 ACF patients and 100 PCF patients were enrolled in the study. Compared to the PCF group, the ACF group had more female (51.9% vs 44.0%), more children with ≤14 years of age (2.8% vs 0%), and more elderly with ≥65 years of age (30.6% vs 23.0%) although these are not statistically significant differences. Median age was slightly higher in ACF than in PCF (55 [IQR 43.8–68] vs 52.5 [IQR 45–62.3], p = 0.556). The proportion of participants who were jobless or were only doing housework was higher in ACF than in PCF (41.7% vs 28.0%). Distance from home to nearest health centre was significantly longer in ACF than in PCF (4km [IQR 3–7] vs 3km [IQR 2–5], p = 0.014). The ACF group had more clinically-diagnosed patients compared to the PCF group (47.2% vs 16.0%, p<0.001).

**Table 1:** Characteristic of study participants by case finding approach.

Characteristics		ACF		PCF		p-value^†^
		N = 108	(%)	N = 100	(%)	
Sex	M	52	(48.1%)	56	(56.0%)	0.320
	F	56	(51.9%)	44	(44.0%)	
Age	Median	55	[IQR: 43.8–68]	52.5	[IQR: 45–62.3]	0.556
	Mean	53.4	[SD: 17.5]	52.1	[SD: 14.4]	
Age group	≤14	3	(2.8%)	0	(0.0%)	0.287
	15–24	4	(3.7%)	4	(4.0%)	
	25–34	14	(13.0%)	9	(9.0%)	
	35–44	7	(6.5%)	11	(11.0%)	
	45–54	24	(22.2%)	31	(31.0%)	
	55–64	23	(21.3%)	22	(22.0%)	
	≥65	33	(30.6%)	23	(23.0%)	
Occupation	Farmer	38	(35.2%)	45	(45.0%)	0.224
	Housework/jobless	45	(41.7%)	28	(28.0%)	
	Private sector	18	(16.7%)	21	(21.0%)	
	Public servant	2	(1.9%)	4	(4.0%)	
	Student	4	(3.7%)	1	(1.0%)	
	Other	1	(0.9%)	1	(1.0%)	
Household income quartile	Low	28	(25.9%)	23	(23.0%)	0.901
	Lower middle	24	(22.2%)	26	(26.0%)	
	Higher middle	29	(26.9%)	25	(25.0%)	
	High	27	(25.0%)	26	(26.0%)	
Distance from home to HC (km)	Median	4.0	[IQR: 3–7]	3.0	[IQR: 2–5]	0.014*
Time required to travel to HC (minutes)	Median	20	[IQR: 10–30]	15	[IQR: 10–22.5]	0.350
Bacteriological status	Bac+	57	(52.8%)	84	(84.0%)	<0.001*
	Bac-	51	(47.2%)	16	(16.0%)	
Type of contact in ACF	Household	9	(8.3%)	NA	NA	NA
	Neighbourhood	99	(91.7%)	NA	NA	

^*^ Significant difference (P<0.05)

^†^ Pearson’s chi-square test for all categorical data (Welch T-test for age comparison)IQR: Interquartile range, SD: Standard deviation, Bac+: Bacteriologically-confirmed TB (Xpert positive for ACF, Smear positive for PCF), Bac-: Clinically-diagnosed TB, HC: Health centre

### Costs before and during TB treatment (Table 2 and 3)

#### Total cost

**Fig 1:**
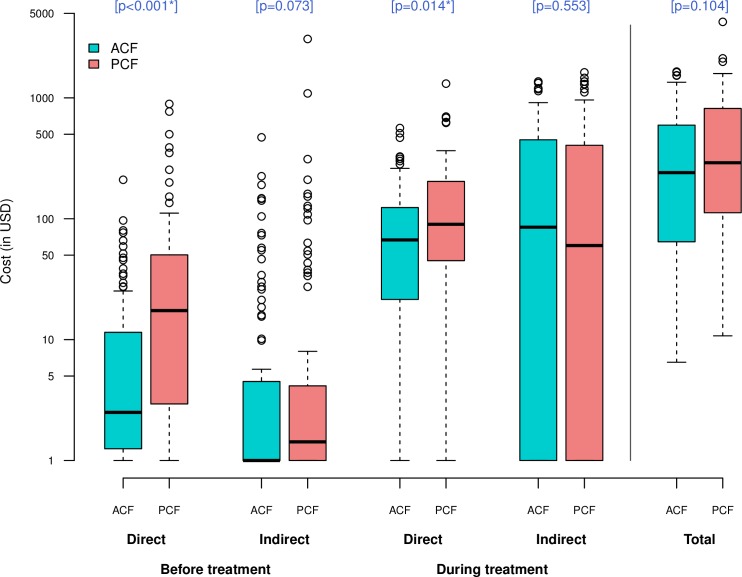
Comparison of cost distribution between ACF and PCF. Box-and-whisker plots indicate the median, 25^th^ and 75^th^ centiles, and the range of values. All values less than one were converted to one in order to use a log scale for the y-axis. *Significant difference (P<0.05).

The total cost incurred during an episode of TB showed a considerable variation in both groups, ranging from $6.5 to $1649 in ACF and from $10.8 to $4251 in PCF. The median total costs were lower by 17% in ACF than in PCF ($240.7 [IQR 65.5–594.6] vs $290.5 [IQR 113.6–813.4], p = 0.104) (Fig 1). Compared to PCF patients, ACF patients incurred significantly lower median costs before treatment ($5.1 [IQR 1.5–25.8] vs $22.4 [IQR 4.4–70.8], p<0.001). Cost before treatment accounted for 7.8% of total costs in ACF and 21.8% in PCF. No clear difference was found in the median cost during treatment between the two groups ($233.2 [IQR 52.4–568.4] vs $235 [IQR 88.3–635.9], p = 0.367). When disaggregated by “direct-indirect” and “before-during”, direct costs both before and during treatment were significantly lower in ACF than in PCF ($2.5 [IQR 1.2–11] vs $14.8 [IQR 2.2–47.3], p<0.001, and $66.8 [IQR 22–122.6] vs $90 [IQR 45–202.5], p = 0.014, respectively) (Fig 1).

**Table 2:** Direct and indirect costs before and during TB treatment by case finding approach (in USD).

Cost category		ACF						PCF						p-value^†^
		Median	[IQR]	Mean	(SD)	[Range]	% total mean	Median	[IQR]	Mean	(SD)	[Range]	% total mean	
Before treatment	Direct	2.5	[1.2–11]	12.4	(26.5)	[0–210]	3.1%	14.8	[2.2–47.3]	57.4	(136.8)	[-9.2–891]	10.7%	<0.001*
	Indirect	0	[0–4.1]	18.6	(58.8)	[0–471.4]	4.7%	1.4	[0–4.1]	59.1	(325.9)	[0–3069]	11.0%	0.073
During treatment	Direct	66.8	[22–122.6]	98.1	(105.9)	[0–562.8]	24.6%	90	[45–202.5]	148.3	(182.4)	[0–1311]	27.7%	0.014*
	Indirect	85.1	[0–450]	269.8	(361.4)	[0–1365]	67.6%	60	[0–382.5]	270.2	(405.3)	[0–1625]	50.5%	0.553
Total		240.7	[65.5–594.6]	399	(416)	[6.5–1649]	100.0%	290.5	[113.6–813.4]	534.9	(627.8)	[10.8–4251]	100.0%	0.104

^*^ Significant difference (P<0.05)

^†^ Wilcoxon rank-sum test

IQR: Interquartile range, SD: Standard deviation. A number with a negative value of less than zero is due to insurance reimbursement.

**Table 3:** Breakdown of direct and indirect costs before and during TB treatment by case finding approach (in USD).

Before/during Tx	Cost category	Sub-category	ACF					PCF					p-value^†^
			Median	[IQR]	Mean	(SD)	[Range]	Median	[IQR]	Mean	(SD)	[Range]	
Before Tx	Direct medical costs	Admin cost	0	[0–0]	0.1	(0.6)	[0–5]	0	[0–0.2]	1.6	(11.3)	[0–112.5]	<0.001*
		Test cost	0	[0–0]	0.7	(2.9)	[0–20]	0	[0–0]	2.3	(7.4)	[0–50]	0.03*
		X-ray cost	0	[0–0]	0.6	(2.4)	[0–15]	0	[0–3.8]	2.1	(4.2)	[0–20]	<0.001*
		Drug cost	0	[0–6.5]	7.6	(19)	[0–135]	6.2	[0–25.5]	36.6	(96.5)	[0–542.5]	<0.001*
		Hospitalization cost	0	[0–0]	0.1	(1)	[0–10]	0	[0–0]	3.7	(16.4)	[0–112]	0.006*
	Direct non-medical costs	Transportation cost	1.2	[0.6–2]	1.9	(2.5)	[0–17.5]	1.8	[1.1–4.9]	3.7	(5.2)	[0–37.5]	<0.001*
		Food cost	0	[0–0.3]	0.4	(1)	[0–5]	0	[0–2.5]	2.1	(6)	[0–37.5]	0.059
		Guardian cost	0.4	[0–1.2]	1.1	(2.4)	[0–20]	1.1	[0–5.2]	6.4	(18.2)	[0–130.4]	<0.001*
		Insurance reimbursement	0	[0–0]	0	(0)	[0–0]	0	[0–0]	1.2	(6.3)	[0–49.2]	0.01*
	Indirect costs	Lost income due to health seeking	0	[0–1.5]	1.6	(4.2)	[0–32.3]	1	[0–2.8]	20.6	(143.9)	[0–1420]	0.009*
		Lost income due to sick leave	0	[0–0]	17	(57.5)	[0–470]	0	[0–0]	38.5	(198.5)	[0–1648]	0.586
	Sub-total		5.1	[1.5–25.8]	31.1	(65.2)	[0–472.1]	22.4	[4.4–70.8]	116.4	(395.1)	[-5.8–3569]	<0.001*
During Tx	Direct medical costs	Hospitalization cost	0	[0–0]	0	(0)	[0–0]	0	[0–0]	2.7	(23)	[0–226.2]	0.143
	Direct non-medical costs	DOT cost (transportation)	0	[0–0]	9.5	(32.6)	[0–252]	0	[0–0]	30.1	(120.2)	[0–1080]	0.192
		Drug pick-up cost (transportation)	15	[0–26.2]	18.8	(23.5)	[0–150]	12	[0–30]	16.8	(17.9)	[0–75]	0.747
		Follow up examination cost (transportation)	0	[0–0.2]	0.9	(2.2)	[0–12]	0	[0–1]	0.8	(1.8)	[0–9.4]	0.826
		Supplemental food cost	45	[0–78.8]	65.7	(86.2)	[0–450]	60	[22.5–150]	92.9	(105)	[0–600]	0.01*
		Guardian and caregiver cost	0	[0–0]	2.4	(10)	[0–84]	0	[0–0]	3.5	(18.7)	[0–179.8]	0.459
		Interest for borrowed money	0	[0–0]	0.8	(3)	[0–21]	0	[0–0]	1.7	(10.5)	[0–100]	0.956
		Insurance reimbursement	0	[0–0]	0	(0)	[0–0]	0	[0–0]	0.2	(2)	[0–20]	0.303
	Indirect costs	Lost income of patient	0	[0–38.2]	132.5	(289.3)	[0–1365]	0	[0–162.5]	193.6	(380.7)	[0–1625]	0.186
		Reduced household activity of patient	0	[0–0]	108.8	(230.2)	[0–900]	0	[0–0]	45.9	(147)	[0–900]	0.019*
		Lost income of guardian/caregiver	0	[0–8.1]	28.6	(125.9)	[0–1200]	0	[0–0]	27.5	(127.9)	[0–960]	0.392
		Value lost due to sold property	0	[0–0]	0	(0)	[0–0]	0	[0–0]	3.2	(32.5)	[0–325]	0.303
	Sub-total		233.2	[52.4–568.4]	368	(385.6)	[5–1618]	235	[88.3–635.9]	418.5	(440.2)	[9–1907]	0.367
All	Total		240.7	[65.5–594.6]	399	(416)	[6.5–1649]	290.5	[113.6–813.4]	534.9	(627.8)	[10.8–4251]	0.104

*Significant difference (P<0.05)

^†^ Wilcoxon rank-sum test

IQR: Interquartile range, SD: Standard deviation, Tx: treatment. A number with a negative value of less than zero is due to insurance reimbursement.

#### Direct medical cost

Before treatment, drug cost accounted for around 80% of the direct medical costs in both groups; the ACF group reported the significantly higher median cost on drug ($0 [IQR 0–6.5] vs $6.2 [IQR 0–25.5], p<0.001). The medians of administrative cost, test cost, X-ray cost and hospitalization cost were zero in both groups and could be considered minor expenses. Yet the highest points of the range in administrative and hospitalization costs exceeded $100 in PCF. During treatment, hospitalization costs were incurred in only two patients in PCF and none in ACF, and no other costs were incurred under direct medical costs in both groups.

#### Direct non-medical cost

Before treatment, the ACF group reported significantly lower median costs for transportation ($1.2 [IQR 0.6–2] vs $1.8 [IQR 1.1–4.9], p<0.001) and guardian ($0.4 [IQR 0–1.2] vs $1.1 [IQR 0–5.2], p<0.001). During treatment, the highest median cost was reported for supplemental food costs in both groups, with a significantly lower cost in ACF ($45 [IQR 0–78.8] vs $60 [IQR 22.5–150], p = 0.01). The frequently-bought supplemental foods included fruits, drinks, fish, and dessert in both groups. The medians and IQRs for transportation costs for DOT were zero in both ACF and PCF (p = 0.192), while those for picking-up drugs at health centres were $15 (IQR 0–26.2) in ACF and $12 (IQR 0–30) in PCF (p = 0.747), being substantial expenses under direct non-medical costs during treatment.

#### Insurance reimbursement

No insurance reimbursement was reported in the ACF group before and during treatment while 6 PCF patients (6%) before treatment and one PCF patient (1%) during treatment received a reimbursement or subsidy for their OOP expenses (direct medical and/or non-medical costs) mainly through a donor-funded programme. 12 ACF patients (11.1%) and 17 PCF patients (17.0%) were registered in the non-insurance Cambodian Health Equity Funds (HEFs) and Subsidy Scheme that provides poor people with transportation and food costs associated with their health-seeking to public health facilities in addition to granting a user fee waiver at government health facilities [31]. Of 29 patients enrolled in the HEFs scheme, 16 (55%) benefited from the free service at government health centres, however, no patients in this study received subsidy or reimbursement for their non-medical costs through this scheme.

#### Indirect costs

Indirect costs accounted for 72.3% of total costs in ACF and 61.5% in PCF. In both groups, indirect costs during treatment made up larger proportions than indirect costs before treatment (ACF: 67.6% vs 4.7%, PCF: 50.5% vs 11.0%). Before treatment, the ACF group incurred, on average, lower income loss due to health-seeking ($1.6 vs $20.6) and lower income loss due to sick leave ($17 and $38.5), compared to PCF. In particular, lost income due to health-seeking was not a main cost contributor in ACF as it accounted for 5% of total costs before treatment whereas it was 17.7% in PCF.

During treatment, lost income of patients ($132.5 for ACF, $193.6 for PCF) and reduced household activity of patients ($108.8 for ACF, $45.9 for PCF) represented substantial expenses, which are followed by lost income of guardian/caregivers ($28.6 for ACF, $27.5 for PCF). There was only one PCF patient that was affected by the value lost due to sold property ($325 lost by selling livestock property).

#### Overall observation

In both groups, the highest mean costs incurred before treatment were lost income due to sick leave and drug cost; these costs accounted for more than 60% of costs before treatment. Likewise, in both group, the highest mean costs incurred during treatment were supplemental food cost, lost income of patients, and reduced household activity of patients; these represented around 80% of costs during treatment.

Most of the mean costs were found to be lower in ACF than in PCF, however reduced household activity of patients was more than doubled in ACF than in PCF. Many of the median costs and IQRs were zero, showing that these mean costs were mainly driven by some of the patients spending extremely high costs.

### Cost as percentage of reported annual household income (Table 4)

**Fig 2:**
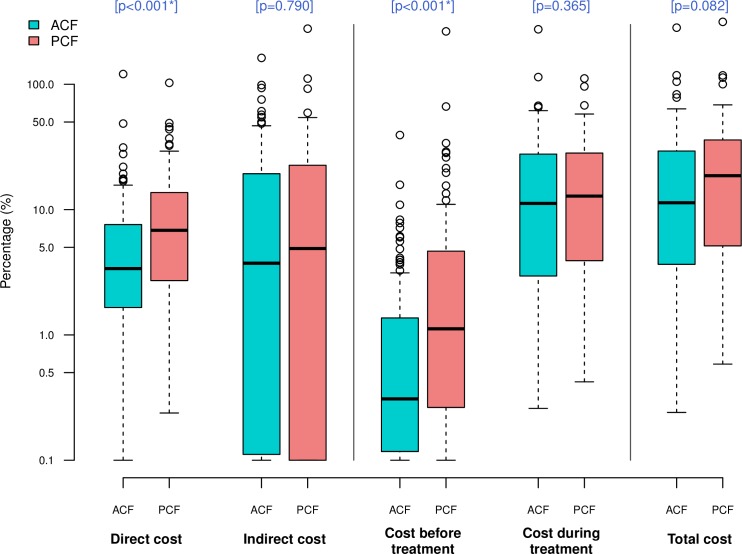
Comparison of cost as percentage of reported annual household income between ACF and PCF. Box-and-whisker plots indicate the median, 25^th^ and 75^th^ centiles, and the range of values. All values less than 0.1 were converted to 0.1 in order to use a log scale for the y-axis. *Significant difference (P<0.05).

Costs as percentage of reported annual household income were consistently lower in ACF than in PCF (Fig 2). The total costs were equivalent to 11.3% (IQR 3.7–29.3) and 18.6% (IQR 5.2–35.7) of annual household income in ACF and PCF respectively (p = 0.082). A financial burden of direct costs on household budget was twice lower in ACF than in PCF (3.4% [IQR 1.7–7.6] vs 6.8% [IQR 2.8–13.6], p<0.001), and that of costs before treatment was more than three times lower in ACF (0.3% [IQR 0.1–1.3] vs 1.1% [IQR 0.3–4.6], p<0.001), showing a statistically significant difference. The same tendency was observed for indirect cost (3.7% [IQR 0.1–19] vs 4.9% [IQR 0.1–22.4], p = 0.79) and costs during treatment (11.2% [IQR 3–27.1] vs 12.8% [IQR 3.7–28.2], p = 0.365) but these were not statistically significant.

**Table 4:** Cost as percentage of reported annual household income.

Breakdown	Category	ACF				PCF				p-value^†^
		Median	[IQR]	Mean	Range	Median	[IQR]	Mean	Range	
	Total costs (%)	11.3	[3.7–29.3]	17.3	[0.2–283.1]	18.6	[5.2–35.7]	21.8	[0.6–314.9]	0.082
Breakdown by cost category	Direct cost (%)	3.4	[1.7–7.6]	4.8	[0–120.7]	6.8	[2.8–13.6]	8.4	[0.2–102.7]	<0.001*
	Indirect cost (%)	3.7	[0.1–19]	12.5	[0–162.4]	4.9	[0.1–22.4]	13.4	[0–277.9]	0.790
Breakdown by timing	Cost before treatment (%)	0.3	[0.1–1.3]	1.3	[0–39.3]	1.1	[0.3–4.6]	4.8	[0–264.4]	<0.001*
	Cost during treatment (%)	11.2	[3–27.1]	15.9	[0.2–274.8]	12.8	[3.7–28.2]	17	[0.4–111.3]	0.365

*Significant difference (P<0.05)

^†^ Wilcoxon rank-sum test. IQR: Interquartile range.

### Catastrophic costs and coping strategies

**Fig 3:**
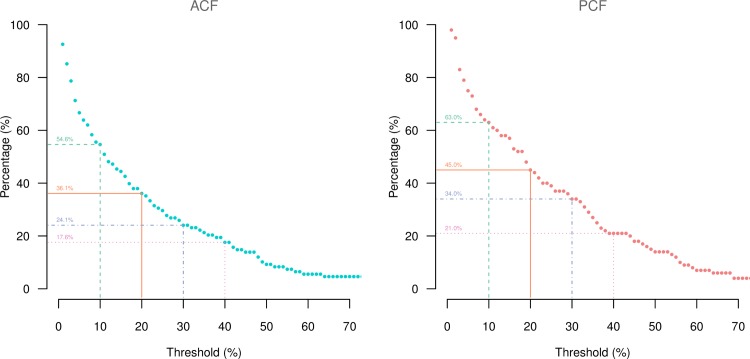
Proportion of patients facing catastrophic costs with different thresholds by case finding approach.

The proportion of patients who spent >10% of annual household income on total costs was lower in ACF than in PCF (54.6% vs 63.0%, p = 0.278) (Fig 3). The same tendency was observed in the comparison with different thresholds of 20% (36.1% vs 45.0%, p = 0.244), 30% (24.1% vs 34.0%, p = 0.154) and 40% (17.6% vs 21.0%, p = 0.655).

**Fig 4:**
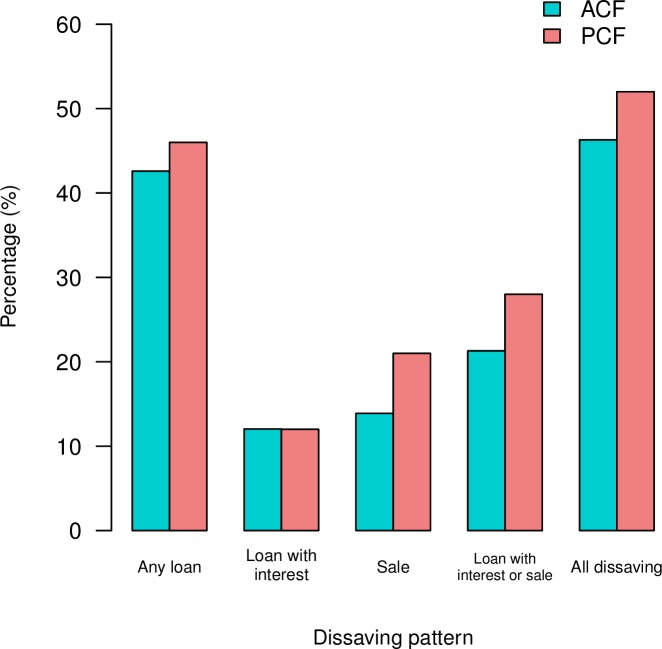
Proportion of patients experiencing dissaving by case finding approach.

The prevalence of dissaving to finance TB-related expenses was found to be low in ACF than in PCF (Fig 4). The largest difference between ACF and PCF was observed for “Sale” (13.9% vs 21%, p = 0.255), which was followed by “Loan with interest or sale” (21.3% vs 28%, p = 0.336), “All dissaving” (46.3% vs 52%, p = 0.494), and “Any loan” (42.6% vs 46%, p = 0.732). However none of them were statistically significant. No difference was found in “Loan with interest” (12% vs 12%, p = 1). Among 36 patients who sold property, 80.6% of them (12 ACF and 17 PCF patients) sold their livestock.

The prevalence of dissaving was investigated by stratifying patients into four categories according to costs as percentage of annual household income of <10%, 10–20%, 20–30% and >30% (Table 5). Cost as percentage of reported household income was significantly associated with “Sale” both in ACF and PCF, with the highest prevalence of “Sale” reported in the highest cost-income band of >30% (26.9% for ACF [p = 0.020], 41.2% for PCF [p = 0.005]). There were no clear demographic differences between different cost-income bands in both groups. When summing up ACF and PCF patients, “Sale” (p<0.001), “Loan with interest or Sale” (p = 0.007) and “All dissaving” (p = 0.019) were significantly associated with cost-income levels.

**Table 5:** Prevalence of dissaving stratified by cost as percentage of annual household income.

Group	Cost as % of household income	No. of patient	Mean cost (USD)	Mean household income (USD)	Mean age	% Male	Any loan			Loan with interest			Sale			Loan with interest or Sale			All dissaving		
							n	%	p-value^†^	n	%	p-value^†^	n	%	p-value^†^	n	%	p-value^†^	n	%	p-value^†^
**ACF**	**<10**	49	82.8	2387	51.6	46.9	17	(34.7)	0.493	5	(10.2)	0.874	2	(4.1)	0.020*	6	(12.2)	0.161	17	(34.7)	0.175
** **	**10–20**	20	401.0	2890	58.7	60.0	10	(50)		3	(15)		4	(20)		6	(30)		11	(55)	
** **	**20–30**	13	486.5	1896	54.4	38.5	6	(46.2)		1	(7.7)		2	(15.4)		3	(23.1)		7	(53.8)	
** **	**>30**	26	949.7	1914	52.3	46.2	13	(50)		4	(15.4)		7	(26.9)		8	(30.8)		15	(57.7)	
**Subtotal**		108	399.0	2307	53.4	48.1	46	(42.6)		13	(12)		15	(13.9)		23	(21.3)		50	(46.3)	
**PCF**	**<10**	37	100.6	2931	52.5	51.4	14	(37.8)	0.441	2	(5.4)	0.261	4	(10.8)	0.005*	6	(16.2)	0.065	15	(40.5)	0.128
** **	**10–20**	18	357.8	2300	49.4	61.1	10	(55.6)		4	(22.2)		1	(5.6)		5	(27.8)		11	(61.1)	
** **	**20–30**	11	562.1	2346	53.9	54.5	4	(36.4)		1	(9.1)		2	(18.2)		2	(18.2)		4	(36.4)	
** **	**>30**	34	1092.5	2060	52.5	58.8	18	(52.9)		5	(14.7)		14	(41.2)		15	(44.1)		22	(64.7)	
**Subtotal**		100	534.9	2457	52.1	56.0	46	(46)		12	(12)		21	(21)		28	(28)		52	(52)	
**Total**	**<10**	86	90.5	2621	52.0	48.8	31	(36)	0.182	7	(8.1)	0.314	6	(7)	<0.001*	12	(14)	0.007*	32	(37.2)	0.019*
** **	**10–20**	38	380.6	2611	54.3	60.5	20	(52.6)		7	(18.4)		5	(13.2)		11	(28.9)		22	(57.9)	
** **	**20–30**	24	521.1	2102	54.2	45.8	10	(41.7)		2	(8.3)		4	(16.7)		5	(20.8)		11	(45.8)	
** **	**>30**	60	1030.6	1997	52.4	53.3	31	(51.7)		9	(15)		21	(35)		23	(38.3)		37	(61.7)	
**Total**		208	464.4	2379	52.8	51.9	92	(44.2)		25	(12)		36	(17.3)		51	(24.5)		102	(49)	

*Significant difference (P<0.05)

^†^ Fisher’s exact test

**Fig 5:**
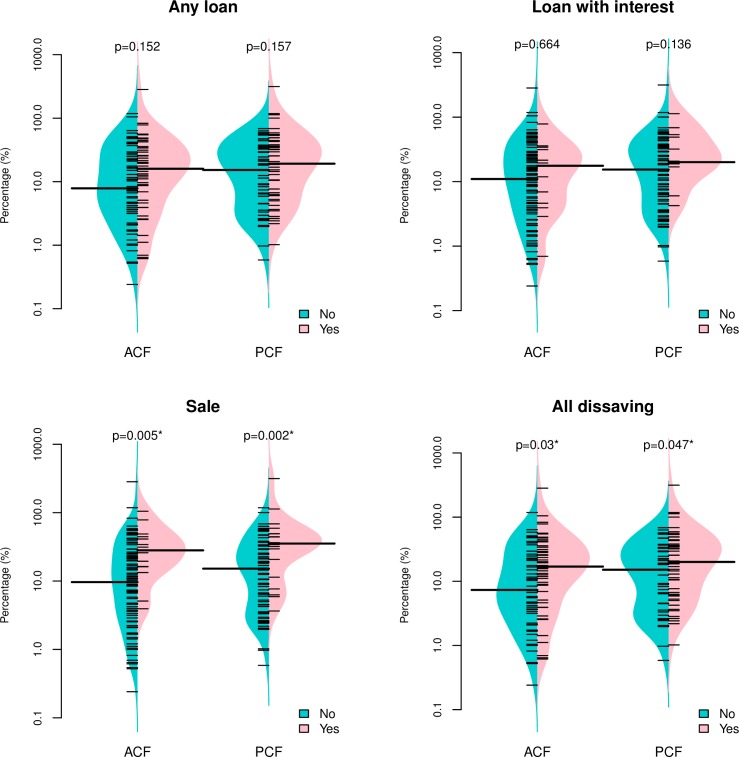
Cost as percentage of annual household income, by dissaving patterns and case finding approach. A short bar indicates an observed value of household’s cost-income levels. A long bar indicates the median. Shaded areas show the density of the distribution. P-values were calculated using a Wilcoxon rank-sum test. A log scale was used for the y-axes.

Median costs as percentage of annual household income were consistently higher in the group with dissaving as compared to the group without dissaving (Fig 5). They were also consistently lower in ACF than in PCF. The statistically significant difference was found between patients with and without “Sale” (p = 0.005 for ACF, p = 0.002 for PCF) and “All dissaving” (p = 0.03 for ACF, p = 0.047 for PCF).

## Discussion

Our study results quantitatively demonstrated lower household costs incurred in ACF patients as compared to PCF patients. In particular, costs before treatment were significantly lower in ACF than in PCF. Similarly, costs as percentage of household income were consistently lower in ACF with significant differences found in direct costs and costs before treatment. Indirect costs constituted the largest portion of total cost. ACF patients were less likely to dissave to afford TB-related costs. Only a limited number of patients received insurance reimbursement.

Why ACF patients incurred lower costs? First, in the Cambodia’s ACF strategy, CENAT actively offered a one-stop shop TB screening service which does not require further repeated visits for patients. Such proactive and tailored approach might reduce diagnostic delays and avoid unnecessary OOP expenditures. For example, in Russia, actively detected patients had the first interaction with a medical health provider one week earlier than passively detected patients [32], showing the contribution of ACF to reduced delays. In the study of Pichenda et al, patients with <1 month treatment delay incurred 8 times lower costs before diagnosis and 1.6 times lower total costs as compared to patients with >3 months treatment delay in Cambodia [29]. This explains the association between shorter delays and lower costs particularly before diagnosis, and supports our argument.

Second, introduction of a rapid sensitive test in ACF, namely Xpert MTB/RIF, in combination with mobile chest X-ray and an onsite clinical assessment greatly increased the diagnostic capacity to detect patients at the early stage of the disease. These patients may typically be asymptomatic or have milder and more chronic symptoms. They therefore might incur lower direct costs due to less-frequent health-seeking visits as well as lower indirect costs due to less sick-leave. In this study, ACF patients were more likely to be bacteriologically-negative (clinically-diagnosed) TB, which could partly explain a less severe disease presentation in ACF patients. This has some similarity with the results from our previous study that Cambodia’s ACF detected more patients with lower smear grade and smear-negative TB [26]. Lower costs incurred for supplemental food and guardian/caregiver during treatment in ACF may be attributable to less severity of the disease in this group.

A concern may be raised about the high proportion of clinically-diagnosed patients in ACF. In Cambodia, the national TB prevalence surveys (conducted in 2002 and 2012) revealed that smear-negative and/or asymptomatic patients were significantly under-diagnosed in the routine case finding [25]. This community-based ACF approach expanded greatly upon traditional approaches to contact investigation mainly to increase case detection in general and partly to fill this diagnostic gap. As a result of the unique target selection, the project detected more patients in children and the elderly as compared to the routine PCF. Such characteristics of ACF participants were likely to have affected the sensitivity of Xpert. The systematic review on diagnostic accuracy of Xpert showed that Xpert achieved an overall pooled sensitivity of 88% (95% Credible interval [Crl], 84–92%) when used as an initial diagnostic test to diagnose pulmonary TB among adults [33]. However the sensitivity decreased to 68% (95% Crl, 61–74%) in people with smear-negative results, and to 66% (95% Crl, 52–77%) in children [33]. One important factor that lowers the sensitivity in children and smear-negatives is bacterial load in the specimen that is generally lower in these populations [34, 35]. Likewise, we assume the sensitivity of Xpert is lower in the elderly given their low quality of sputum specimens that is reported elsewhere [36–38]. In such cases, it may be reasonable to fill this diagnostic gap by facilitating clinical diagnosis based on CXR and clinical findings. Indeed, our data showed that nearly 70% of clinically-diagnosed patients in ACF were either children with ≤14 or older people with ≥ 55. Some might be over-diagnosed but we consider it as an acceptable level given this epidemiological context.

Third, demographic differences between ACF and PCF patients could have resulted in the cost difference. In this study, the ACF group had more female, more children and elderly, and more patients who were house-worker/jobless. Most of them have less or no regular income and more household work, as compared to an economically active population. This most likely caused lower income loss as well as higher cost of reduced household activity in ACF, which could consequently lowered the overall indirect costs in ACF.

The above factors could collectively explain lower costs incurred in ACF. It is often anticipated that ACF detects patients earlier in terms of time therefore reduces only costs before treatment. However, less severity of the disease in ACF may bring prolonged financial benefits for ACF patients over the course of treatment and possibly after treatment completion. Such benefits were more evident in direct costs than in indirect costs perhaps due to the difference in the nature of the two costs that OOP direct expenditure is more responsive to severe patient’s conditions while indirect costs can be triggered even by mild conditions.

In this study, the proportion of patients facing total costs corresponding to >10% of annual household income was 63% in PCF. This was similar to the percentage reported in other countries; 65% in Peru [14], 66–75% in studies from sub-Saharan Africa [39, 40] and 67.7% in China (for patients who complied with treatment) [15], providing a common ground that TB-related cost has the substantial impact on their household budget in low- and middle- income countries. Our results showing the consistently lower proportions found in ACF suggested that ACF has the large potential to reduce the incidence of catastrophic expenditure.

Can dissaving be a proxy for catastrophic costs? Madan et al found a significant positive association between the occurrence of dissaving and total costs incurred, and highlighted the potential of using dissaving as a proxy for catastrophic costs [16]. In our study, we found a significant association between the cost as percentage of annual household income and occurrence of “Sale” both in ACF and PCF using two different analytical approaches. Although “All dissaving” also showed a significant association, the strength of association appeared to be increased by the effect of “Sale” given the relatively disperse distribution of patients with “Any loan” and “Loan with interest” across the percentage scale in Fig 5. In the context of Cambodia, therefore the occurrence of selling property of the household can be a more useful proxy indicator, and this information can be easily collected and monitored using existing standardized recording and reporting forms with a slight modification. However it is important to note that, in this study, more than one third (34.3%) of patients without “Sale” still experienced the total cost that was above 20% of their household income, and in contrast, one in five (19.4%) patients with “Sale” incurred less than 10% of their household income. This implies that the occurrence of “Sale” is not sensitive enough be a close proxy or direct measure for catastrophic expenditure. Further exploration may be needed on how to make the dissaving information more useful for example by combining with other patient factors to better monitor financial risk of TB-affected households.

In this study, the proportions of TB-affected household experiencing “Sale” were 13.9% in ACF, 21% in PCF and 17.3% for both groups. Previous studies from other countries reported a wide range of proportions; 37% in Ghana [41], 19% in Dominican Republic [41], 15.9% in Thailand (patients with income below poverty line) [42], 5% in Vietnam [41], 45% in China [43]. Although available data is limited, dissaving patterns and its implication seem greatly vary across countries, and perhaps the association between dissaving and catastrophic costs may also be context- and culture-specific even within a country. This needs to be carefully taken into account when using dissaving as a potential indicator.

This study has several limitations. First, patient recall bias may be present as we selected all PCF patients who were registered before the ACF session date in each health centre. We tried to minimize the bias by limiting the sampling timeframe to “within four months prior to ACF”, however PCF patients might have more difficulty to recall the details of their expenses and care seeking as compared to ACF patients, which might have influenced the results. Second, we did not sample patients with unfavourable treatment outcomes, drug-resistant TB and/or human immunodeficiency virus infection. Given that they are more likely to experience catastrophic costs due to TB [9, 15], the results presented here might not capture a full picture of TB-related costs. Third, as is common in any cost studies, some of the cost might be over- and/or underestimated. In particular, we carefully assessed indirect costs including reduced household activity of patients and lost income of guardians/caregivers, which may not be included in other cost studies. This might have led to overestimation of indirect costs and/or made the cost data not comparable with other studies. Extrapolating part of health-seeking time for some participants and the assumption of the average working hours/days might have reduced the accuracy of calculating lost income before treatment. Fourth, our relatively small sample size was unable to detect statistical differences in some demographic variables between the two groups, weakening our arguments. It is, however, noteworthy that an analysis of full project database with screening data for more than 70,000 participants has confirmed a high participation of children, female and the elderly.

Our study provided an important policy implication. In this study, only a small number of patients in PCF received reimbursement that covered a part of direct costs. This clearly illustrated the current situation of health financing mechanisms in Cambodia that costs incurred due to TB are predominantly derived from OOP spending by individual care seekers. In our study setting, the HEFs scheme has been playing a certain role in removing financial barriers at the point of care for the poor. However this pro-poor financial protection mechanism has been under-utilized for many eligible patients who seek care in private facilities. Continued efforts are required for increasing the quality and credibility of government health services to maximize its utilization, as well as for expanding sustainable public-private mix approach in TB control, which helps avoid unnecessary OOP spending. In this study, the HEFs scheme was not used for reimbursement of non-medical costs. This could be attributable to the difference in benefit packages or its criteria across different ODs, which needs verification in the field. Given that inadequacies of the HEFs scheme have been pointed out also in the context of non-communicable diseases [44, 45], wider policy discussion is needed on how the scheme can be expanded by capturing all of the core demands of the poor.

In this manner, strengthening traditional approaches to improve the diagnostic pathway and pursuing the best use of existing schemes are indispensable to minimize OOP spending. However, as is found in other countries [9], the largest financial burden due to TB in Cambodia comes from indirect cost (mainly lost income) which is not usually covered by existing social health protection schemes for the informal-sector workers (HEFs and Community-based Health Insurance schemes) [46]. Although Cambodia has three established national social security schemes with an income replacement benefit targeting the formal-sector workers (civil servants, veterans, and employees in private sector) [47], none of the study participants benefited from them for their TB illness. This could indicate a low coverage of these schemes in the study population, likely due to many informal-sector workers in TB patients. Currently, the country is moving toward the establishment of the National Social Health Protection Fund targeting informal-sector workers by incorporating the HEFs and other demand-side financing schemes [46]. For this new scheme to meet the greatest needs of TB-affected households, ensuring an income replacement or similar benefits at the household level will be critical.

At the same time, programmatic efforts are also required to tailor and package pro-poor and proactive case finding intervention strategies in order to detect vulnerable patients as early as possible before considerable costs occur. The Cambodia’s ACF strategy could be the first evidence-based ACF that can help mitigate both direct and indirect costs.

## Conclusions

Our study quantitatively demonstrated the financial hardship of TB-affected household in the routine PCF setting in Cambodia and highlighted the great potential of ACF in mitigating the costs incurred particularly before treatment. Future health policy dialogue should consider how best to design and expand the social protection scheme that can replace lost income for TB-affected households. This will compensate for the most devastating costs in TB, help achieve better health outcomes, and eventually prevent further impoverishment in rural poor communities. Measuring their financial risk using an appropriate and practical indicator is the key for future planning and implementation of the WHO End TB Strategy. An occurrence of selling household property to finance TB-related expenses can be a useful proxy for catastrophic cost in Cambodia.

## Supporting Information

S1 File Questionnaire in English. (PDF)

S2 File Questionnaire in Khmer. (PDF)
