# Supplementary material for: Mitigating Financial Burden of Tuberculosis through Active Case Finding Targeting Household and Neighbourhood Contacts in Cambodia
Source: PLoS One. 2016 Sep 9;11(9):e0162796. doi: 10.1371/journal.pone.0162796 (PMC5017748; doi:10.1371/journal.pone.0162796)
Supplement: S1 File — (PDF) [file pone.0162796.s001.pdf]

Participant code:_______________

1	General Information
**1.1** Interviewer Name	☐ Chamroen ☐ Fanita ☐ Faramy
**1.2** Date (dd.mm.yy)	
**1.3** Village	
**1.4** Health centre	
**1.5** Operational District	

2	Patient Information (to be filled in by interviewer with the help of TB&Lab register)
**2.1** Gender ☐ Male ☐ Female	**2.2** Age of Patient ……………
**2.3** Type of TB	☐ Bacteriological positive ☐ Bacteriological negative
**2.4** Total duration of treatment	☐ 6 months ☐ 8 months ☐ Other(specify): ________
**2.5** Treatment Regimen	☐ New Pulmonary ☐ Retreatment
**2.6** Interviewee	☐ Patient ☐ Guardian — If Guardian... ☐ Parent ☐ Extended Family ☐ Grandparent ☐ Other(specify): ________
**2.7** HIV status (only if indicated)	☐ Positive ☐ Negative ☐ Not tested ☐ Unknown ☐ Declined

**2.8**	Participant Category		
**a1** ☐ **ACF** (☐ select Household or ☐ Neighborhood)		**b1** ☐ **PCF**	
**a2** HC registration number:		**b2** HC registration number:	
**a3** OD registration number:		**b3** OD registration number:	
	(dd.mm.yy)		(dd.mm.yy)
**a4** Date of receiving ACF information		**b4** Date of smear examination	
**a5** Date of ACF Session		**b5** Date of Xray result	
**a6** Date of registration at HC		**b6** Date of registration at HC	
**a7** Date treatment started		**b7** Date treatment started	
**a8** Date of treatment completion		**b8** Date of treatment completion	

**2.9** Marital Status	☐ Married ☐ Single ☐ Divorced/separated ☐ Widowed
**2.10** Residence	☐ Urban ☐ Suburban ☐ Rural ☐ Homeless/displaced
**2.11** Phone number of patient	

3	Health Seeking

3.1 Did you have any TB symptoms before diagnosis? (If no go to question 3.3) ☐ Yes ☐ No

3.2 How long did you experience these symptoms before TB treatment initiation? When did it start?(dd.mm.yy)

Cough	☐ Yes	☐ No	_______ days	Date of onset:	__________
Coughing up blood (Haemoptysis)	☐ Yes	☐ No	_______ days	Date of onset:	__________
Sputum	☐ Yes	☐ No	_______ days	Date of onset:	__________
Night sweats	☐ Yes	☐ No	_______ days	Date of onset:	__________
Weight loss	☐ Yes	☐ No	_______ days	Date of onset:	__________

Chest pain ☐ Yes ☐ No _______ days Date of onset: ______________

Fatigue ☐ Yes ☐ No _______ days Date of onset: ______________

Fever ☐ Yes ☐ No _______ days Date of onset: ______________

Other (specify): ______________ ☐ Yes ☐ No _______ days Date of onset: ______________

3.3 Did you seek treatment or advice for these symptoms at any of the following?
(check all that apply)
Where did you first go? (circle first place of treatment)

Government Hospital ☐ Yes ☐ No Health Center ☐ Yes ☐ No

Private hospital/clinic ☐ Yes ☐ No Pharmacy, drug & grocery store ☐ Yes ☐ No

Traditional healer ☐ Yes ☐ No Other(specify): ______________ ☐ Yes ☐ No

3.4 How far is the nearest government facility for

a TB diagnosis and treatment _______ minutes walking _______ minutes with transport _______ Distance (Km)

b TB treatment only _______ minutes walking _______ minutes with transport _______ Distance (Km)

Section 4: Please fill out separate sheet and provide detail information on Pre diagnosis & diagnosis costs

5	Treatment Costs

Costs related to DOT

5.1 Where did you take your TB drugs?

☐ Health facility/hospital ☐ Home ☐ Community ☐ Workplace ☐ Dispensary

5.2 a. How many times per week did you have to go there to take your drugs? ______________ times per week

b. How long did you have to go there to take your drugs? Duration: ______________ weeks

5.3 How long did it take you to get there (one way)?

_______ minutes walking _________ minutes with transport Other(specify): _________

5.4 How long did one of these visits take on average, including time on the road and waiting time? _______ minutes

5.5 From your home to the DOT place (selected in 5.1), how much did it cost if you took transport? (both ways)

______________ Riel

5.6 How much did you spend on food? On the road, while waiting, for lunch? ______________ Riel

Costs related to ☐ picking up TB drugs or ☐ drug delivery

5.7 a. How often did you travel to the health facility/ hospital to pick up your TB drugs? ___________ Times / month

b. How long did you travel there to pick up your TB drugs? Duration: ___________ months

5.8 How long did it take you to get there (one way)?

_________ minutes walking ___________ minutes with transport Other(specify): ________________

5.9 How long did one of these visits take on average, including time on the road and waiting time? ____________ minutes

5.10 From your home to the facility, how much did it cost if you took transport? (both ways) ________________ Riel

5.11 If you went to a facility to pick up your TB drugs, how much did you spend on food? On the road, while waiting, for lunch? ____________ Riel

5.12 Did you have to pay administration fees or delivery when picking up your TB drugs? ☐ Yes ☐ No

a If yes, how much? __________________ Riel

5.13 Did you have any accommodation costs when picking up your TB drugs? ☐ Yes ☐ No

a If yes, how much? __________________ Riel

5.14 Did you pay for TB drugs? ☐ Yes ☐ No

a If yes, how much? __________________ Riel

Costs related to follow up tests

5.15 Did you ever have to go to the health facility in addition to your regular visits for follow up tests since the beginning of your treatment? (If no go to question 6.1) ☐ Yes ☐ No

a If yes how many times? ____________ times

5.16 If yes, did you have to pay any additional costs at any time of these visits? ☐ Yes ☐ No

a If so, what kind of costs and how much per visit?

Fees: _________________ Sputum Test: ___________________ X-ray: _______________

Other: ________________________ Total: _______________

5.17 How long did one of these follow-up visits take on average, including time on the road, waiting time and tests? (total turnaround time) _________________ minutes

5.18 How much did you spend on transport, food and accommodation for each visit on average?

Transport: ______________ Food: _______________ Accommodation: ____________

6 Guardian Costs

6.1 If any family/friend/DOT supporter accompany you on any visit during TB treatment / follow-up visit/picking up TB drugs / and coming to your place for DOT, did they pay additional cost for their visits? (If no go to question 7.1) ☐ Yes ☐ No

a If yes, on how many visits did your family/friend/DOT supporter accompany you or go in your place?

Treatment/follow-up visit: ______________ times

Costs during treatment per visit: Transport: _____________ Food: _______________ Accommodation: ___________

Total treatment ______________ Riel

6.2 How much does your family/friend/DOT supporter earn per day?

______________ Riel ☐ Doesn't earn

6.3 Why did someone accompany you? (check all that apply)

☐ Distance ☐ Security ☐ Administrative barriers ☐ Too ill to travel alone
☐ Was required for treatment ☐ Patient was a minor ☐ Other (specify) ____________

7 Hospitalization Costs

7.1 Were you hospitalized during your TB treatment? (If no go to question 8.1) ☐ Yes ☐ No

a If yes, how many days in total did you stay in hospital? ____________ Days

7.2 How much did you pay in the hospital per day?

Bed price: ____________

Food (not provided by hospital): ____________ Transport (return): ____________

Drugs* ____________ Tests** ____________ Other (specify): ____________

* Do not repeat TB drug cost described in 5.14
** Do not repeat test costs described in 5.16 TOTAL: ____________ Riel + administration fees: ____________ Riel

7.3 Did any family/friend STAY with you while in hospital? ☐ Yes ☐ No

a If yes, How many days did he/she stay with you (sleep there)? ____________ Days

7.4 Were there any extra costs for your family/friend for staying at the hospital? ☐ Yes ☐ No

Accommodation (hospital or other): ____________ Food: ____________

Transport: ____________ Other (specify): ____________ TOTAL: ____________

7.5 How much does your family/friend earn per day?

____________ Riel ☐ Doesn't earn

7.6 Did any OTHER family/friend visit you in hospital? (If no go to question 8.1) ☐ Yes ☐ No

a If yes, how many times of visit? ________ Times

b How much did they pay for each visit?

Accommodation (hospital or other): ____________ Food: ____________

Transport: ____________ Other (specify): ____________ TOTAL: ____________ Riel

c How long were the visits including travel time? ____________ minutes

8 Other Costs Food Supplements

8.1 Did you buy any supplements for your diet because of TB illness, for example, vitamins, meat,energy drinks, soft drinks, fruit or medicines? (If no go to question 9.1) ☐ Yes ☐ No

a If yes, What kind of items? (specify) (check all that apply)

☐ Fruits ☐ Drinks ☐ Vitamins/Herbs ☐ Meat ☐ Other (specify): ____________

8.2 How much did you spend on these items per month on average? ____________ Riel

9 Other Illnesses

9.1 Do you have any chronic illness for which you are receiving treatment? (If no go to question 9.3)

☐ Only before TB ☐ Only after TB ☐ Before & after TB ☐ No

a If yes, Which? a ____________ b ____________ c ____________

9.2 Were there any additional costs for you because of this other illness BESIDES the costs that you have already mentioned? ☐ Yes ☐ No

a If yes, how much were these additional costs on average per month?

Tests: _______ Drugs:_______ Food: _______ Transport: _______ Other: __________ TOTAL: _______

9.3 How much did you spend on healthcare on average per month BEFORE the TB illness? ______________Riel

9.4 How much do you spend on healthcare on average per month NOW? ______________Riel

10 Reimbursement/ Equity Card

10.1 Do you have any kind of private or governmental health/medical insurance scheme? (If no go to question 10.2)

☐ Yes ☐ No

a If yes, what type?

☐ Reimbursement scheme ☐ Monthly medical allowance ☐ Equity card ("poor card")

☐ Family/community fund ☐ Western scheme (contract) ☐ Donor

☐ Other(specify): __________________________

10.2 Do you know about the equity card system in Cambodia? ☐ Yes ☐ No

10.3 Have you received reimbursement for any costs related to TB treatment?* ☐ Yes ☐ No

(If reimbursement for pre-diagnosis/diagnosis, use Section 4)

a How much have you received in reimbursement? ____________________ Riel

11 Coping Costs

11.1 Did you borrow any money to cover costs due to TB illness? (If no go to question 11.2) ☐ Yes ☐ No

a If yes, how much did you borrow? _______________ Riel

b From whom did you borrow? ☐ Family ☐ Neighbors/friends ☐ Private bank

(check all that apply) ☐ Cooperative ☐ Other (specify) ______________________

c What is the interest rate on the loan? (%)

_____________ % ☐ I do not pay interest ☐ I'm not expected to pay back the money

11.2 Have you sold any of your property to finance the cost of TB illness? ☐ Yes ☐ No

If yes, what did you sell? (check all that apply)

a ☐ Land ☐ Livestock ☐ Transport/vehicle ☐ Household item ☐ Farm produce

☐ Other (specify)

b What is the estimated market value of the property you sold? _________________________ Riel

c How much did you earn from the sale of your property? _______________ Riel

12 Socioeconomic Information Individual Situation and Income

12.1 Who is the primary income earner in the household?

☐ Patient ☐ Wife/mother ☐ Husband/father ☐ Extended family ☐ Son/daughter

☐ Other (specify): _______________

Participant code:_______________

12.2 What is the highest level of education of…?

a The patient

☐ Illiterate ☐ Primary ☐ Secondary ☐ Graduate/certificate ☐ Other(specify): __________

b The primary income earner?

☐ Illiterate ☐ Primary ☐ Secondary ☐ Graduate/certificate ☐ Other(specify): __________

c Head of household?

☐ Illiterate ☐ Primary ☐ Secondary ☐ Graduate/certificate ☐ Other(specify): __________

12.3 Were you formally employed before diagnosis?

☐ Yes, formal work ☐ On sick leave ☐ Schooling ☐ Housework

☐ No, informal work ☐ Retired ☐ Other(specify): __________

12.4 Were you formally employed during treatment?

☐ Yes, formal work ☐ On sick leave ☐ Schooling ☐ Housework

☐ No, informal work ☐ Retired ☐ Other(specify): __________

a Is the reason for not working related to TB illness? ☐ Yes ☐ No

b If yes, when was the last time you were working? Date (mm/yy) __________

12.5 Are you currently formally employed?

☐ Yes, formal work ☐ On sick leave ☐ Schooling ☐ Housework

☐ No, informal work ☐ Retired ☐ Other(specify): __________

a Is the reason for not working related to TB illness? ☐ Yes ☐ No

b If yes, when was the last time you were working? Date (mm/yy) __________

12.6 How are you usually paid?

☐ Cash ☐ In kind ☐ Cash and in kind ☐ Not paid

☐ Bank transferred salary ☐ Other(specify): ____________________

12.7 What was your personal take home earning per week on average BEFORE TB illness?
or other social support)

______________ Riel per week ☐ Didn't earn

12.8 What was your personal take home earning per week on average DURING TB illness?
(includeswelfare, disability,or other social support)

______________ Riel per week ☐ Didn't earn

a If the answer differs from 12.7, Is the change related to TB illness? ☐ Yes ☐ No

12.9 What is your personal take home earning per week on average NOW?
(includes, welfare,disability, or other social support)

______________ Riel per week ☐ Didn't earn

a If the answer differs from 12.7, Is the change related to TB illness? ☐ Yes ☐ No

12.10 Have you ever stopped working/going to school/doing housework due to TB? ☐ Yes ☐ No

a If yes, for how long? ______ Months

12.11 Did someone stay home **SPECIFICALLY** to take care of you due to TB? ☐ Yes ☐ No

a If yes, for how long? __________ Weeks

b Did they quit or reduce their income-earning job to stay home and care for you? ☐ Yes ☐ No

c If yes, how much did he/she lose income per weeks on average? __________________ Riels

12.12 How regularly did you work before you became ill with TB

☐ Throughout the year ☐ Seasonal/part of year ☐ Day labour ☐ Other(specify) ______

12.13 Did you have to change jobs when you became ill with TB? ☐ Yes ☐ No or N/A

12.14 What is your main occupation?

☐ Sales/service ☐ Farmer ☐ Household ☐ Production/construction worker

☐ Student ☐ Jobless ☐ Public servant ☐ Other(specify) __________

12.15 How many hours did you work on average per day…?

a BEFORE you became ill with TB? ________ Hours

b DURING treatment? ________ Hours

c AFTER treatment? ________ Hours

12.16 If answers different, was the change related to TB illness? ☐ Yes ☐ No

a If answer differs, Is someone doing the work that you used to do? (check all that apply)

☐ Daughter ☐ Son ☐ Spouse ☐ Friend ☐ Nobody ☐ Other family

12.17 Do you have children of or below school age? (If no go to question 12.19) ☐ Yes ☐ No

12.18 Did all of your children of school age attend school regularly during your treatment? ☐ Yes ☐ No

a If no, why not? Tick most appropriate

☐ Needs to help around the house ☐ No money for school fees ☐ Also sick

☐ Has to work to earn income ☐ Other (specify) __________

b Did any of your children of or below school age work to finance costs due to TB illness? ☐ Yes ☐ No

12.19 If you employed someone to do the housework for your household, how much would it cost?

a While you were sick? ____________ Riel per day

b While you are healthy? ____________ Riel per day

12.20 Are you financially independent? ☐ Yes ☐ No

12.21 Has TB illness affected your social or private life in any way? (check all that apply)

☐ No ☐ Divorce ☐ Loss of job ☐ Dropped out of school ☐ Sick child

☐ Separated from spouse/partner ☐ Disruption of sexual life ☐ Other (specify) ________

12.22 Has TB illness resulted in a financial burden? ☐ Yes ☐ No

13 Household Income and Spending

13.1 How much do you estimate was the average income of your household per month BEFORE TB illness?
(for all persons in the house, including patient, welfare, government or social assistance)

Income patient: __________ Income rest of household: __________ Welfare payments: __________

Government assistance: __________ Other(specify): ______________ TOTAL: __________ Riel

13.2 How much do you estimate was the average income of your household per month DURING treatment?

Income patient: ____________ Income rest of household: ____________ Welfare payments: ____________

Government assistance: ____________ Other(specify): ____________________ TOTAL: ____________ Riel

13.3 How much do you estimate was the average income of your household per month AFTER TB illness?

Income patient: ____________ Income rest of household: ____________ Welfare payments: ____________

Government assistance: ____________ Other(specify): ____________________ TOTAL: ____________ Riel

13.4 How many people regularly sleep in your house? (including patient) _______ Persons

13.5 How many of the household members are paid for working? (include patient, and payment in kind or farm produce) _______ Persons

13.6 Besides yourself, did anyone else of your household receive treatment for TB? ☐ Yes ☐ No

a If yes, how many? _______ Persons

13.7 How much food did your household consume every month on average BEFORE, DURING and AFTER TB illness?

(If home production) If the food that you consumed per month before/during/after TB illness was sold on the market: How much would it be worth? (plus how much you spent on average on food not produced at home?)

a BEFORE: __________ Riel

b DURING: __________ Riel **b2.** if answer differs from a, the change is due to TB? ☐ Yes ☐ No

c AFTER: __________ Riel **c2.** if answer differs from a, the change is due to TB? ☐ Yes ☐ No

14 Socioeconomic Indicators

14.1 What is your main source of drinking water?

☐ Rainwater ☐ Lake/pond/river ☐ Private well/bore hole

☐ Piped water ☐ Bottled water ☐ Public water supply

14.2 What type of toilet facility is available?

☐ No facility/bush/field ☐ Shared pit toilet ☐ Own latrine ☐ Flush toilet

14.3 How many rooms are there in your house?

☐ 1 room ☐ 2 rooms ☐ 3 rooms ☐ 4 or more rooms

a Do you own the house or residence you live in? ☐ Yes ☐ No

14.4 Do you own? Tick all that apply

☐ Radio ☐ Television ☐ Mobile phone ☐ Non-mobile phone ☐ Fridge

☐ Wardrobe ☐ Sewing machine ☐ CD/DVD player ☐ Generator

14.5 If the government could provide you with some service to ease the burden of TB on you and your household, what would you prefer to have? Choose only one

☐ Transport voucher ☐ Food voucher ☐ More efficient service

☐ Other (specify): ____________________

14.6 How much would you be willing to pay for not becoming ill with TB in the first place?

☐ Less than $20 ☐ $20-50 ☐ $51-100 ☐ More than $100

Comments of interviewer

Signature

About how much did you spend for each of these visits before you were diagnosed with TB, including the visit when you actually received your diagnosis?*

For all that don't apply, mark N/A. Fill one line per visit. Fill in chronological order.

	A	B	C	D	E	F	G	H	I	J	K	L	M
Visit	Type of provider**	Date of visit***	Distance (Km)	Total Time Spent per visit (in hours, includes travel time)	Admin Cost/ Consultative registration	Test Cost (for sputum or other except x-ray)	X-ray Costs (includes sending x-rays to radiologist, travel & fees)	Drug Costs (all kinds total)	Travel Costs (round trip)	Food Costs (total)	Accommodation/ hospitalization Costs (total)	Sub-Total Costs per visit	insurance Reimburseme nt
1				T:					Pt:	Pt:	Pt:	Pt:	
				O:					G	G	G	G	
2				T:					Pt:	Pt:	Pt:	Pt:	
				O:					G	G	G	G	
3				T:					Pt:	Pt:	Pt:	Pt:	
				O:					G	G	G	G	
4				T:					Pt:	Pt:	Pt:	Pt:	
				O:					G	G	G	G	
5				T:					Pt:	Pt:	Pt:	Pt:	
				O:					G	G	G	G	
6				T:					Pt:	Pt:	Pt:	Pt:	
				O:					G	G	G	G	
7				T:					Pt:	Pt:	Pt:	Pt:	
				O:					G	G	G	G	

8				T:					Pt:	Pt:	Pt:	Pt:	
				O:					G	G	G	G	
9				T:					Pt:	Pt:	Pt:	Pt:	
				O:					G	G	G	G	
Total Direct Prediagnosis and Diagnostic costs (sum sub-totals) minus insurance =											Riel		

* This includes travel to ACF camp as well (If ACF patient, select "6" for "Type of provider")

** 1, Government hospital 2, Health center 3, Private hospital/clinic 4, Pharmacy, drug & grocery store
5, Traditional healer 6, Community-based TB screening (ACF) 7, Others (specify in the box)

*** This is very important for this reserach.
