# Supplementary material for: Mitigating Financial Burden of Tuberculosis through Active Case Finding Targeting Household and Neighbourhood Contacts in Cambodia
Source: PLoS One. 2016 Sep 9;11(9):e0162796. doi: 10.1371/journal.pone.0162796 (PMC5017748; doi:10.1371/journal.pone.0162796)
Supplement: S2 File — (PDF) [file pone.0162796.s002.pdf]

លេខកូដអ្នកចូលរួម:________________

1	ព័ត៌មានទូទៅ
1.1 ឈ្មោះអ្នកសម្ភាសន៍	☐ ចំរើន ☐ ហ្វានីតា ☐ ហ្វារ៉ាមី
1.2 កាលបរិច្ឆេទ (ថ្ងៃ ខែ ឆ្នាំ)	
1.3 ភូមិ	
1.4 មណ្ឌលសុខភាព	
1.5 ស្រុកប្រតិបត្តិ	

2	ព័ត៌មានអំពីអ្នកជំងឺ<br>(ត្រូវបំពេញដោយអ្នកសម្ភាសន៍ដោយមានជំនួយពី TB & Lab register)		
2.1 ភេទ ☐ប្រុស ☐ស្រី		2.2 អាយុអ្នកជំងឺ ___________	
2.3 ប្រភេទជំងឺរបេង	☐ មានមេរោគ (b+) ☐ គ្មានមេរោគ (b-)		
2.4 រយៈពេលព្យាបាលសរុប	☐ ៦ ខែ ☐ ៨ ខែ ☐ ផ្សេងៗ (បញ្ជាក់)៖ ___________		
2.5 វិធីសាស្ត្រព្យាបាល	☐ របេងក្នុងសួតរកឃើញថ្មី ☐ ការព្យាបាលសារឡើងវិញ		
2.6 អ្នកផ្តល់សម្ភាសន៍	☐អ្នកជំងឺ<br>☐អាណាព្យាបាល	**ប្រសិនបើអាណាព្យាបាល...**	☐ឪពុកម្តាយ ☐គ្រួសារសាច់ឆ្ងាយ<br>☐យាយតា ☐ផ្សេងៗ(បញ្ជាក់)៖ ________
2.7 ស្ថានភាពជំងឺអេដស៍<br>(ប្រសិនបើមានប៉ុណ្ណោះ)	☐វិជ្ជមាន ☐អវិជ្ជមាន ☐ពុំបានធ្វើតេស្ត ☐មិនដឹង<br>☐បដិសេធ		

2.8	**ប្រភេទអ្នកចូលរួម**		
ក1 ☐ ACF (☐គ្រួសារ ឬ ☐អ្នកជិតខាងដែលបានជ្រើសរើស)		ខ1 ☐ PCF	
ក2 លេខចុះឈ្មោះនៅ HC៖		ខ2 លេខចុះឈ្មោះនៅ HC៖	
ក3 លេខចុះឈ្មោះនៅ OD៖		ខ3 លេខចុះឈ្មោះនៅ OD៖	
	(ថ្ងៃ ខែ ឆ្នាំ)		(ថ្ងៃ ខែ ឆ្នាំ)
ក4 កាលបរិច្ឆេទទទួលព័ត៌មាន ACF		ខ4 កាលបរិច្ឆេទពិនិត្យឡាម	
ក5 កាលបរិច្ឆេទនៃវគ្គ ACF		ខ5 កាលបរិច្ឆេទលទ្ធផល Xray	
ក6 កាលបរិច្ឆេទចុះឈ្មោះនៅ HC		ខ6 កាលបរិច្ឆេទចុះឈ្មោះនៅ HC	
ក7 កាលបរិច្ឆេទចាប់ផ្តើមការព្យាបាល		ខ7 កាលបរិច្ឆេទចាប់ផ្តើមការព្យាបាល	
ក8 កាលបរិច្ឆេទបញ្ចប់ការព្យាបាល		ខ8 កាលបរិច្ឆេទបញ្ចប់ការព្យាបាល	
2.9 ស្ថានភាពគ្រួសារ ☐ រៀបការ ☐ នៅលីវ ☐ លែងលះ/រស់នៅបែកគ្នា ☐ ពោះម៉ាយ/មេម៉ាយ			
2.10 ទីលំនៅ	☐ ទីក្រុង ☐ ជាយក្រុង ☐ ជនបទ ☐ គ្មានផ្ទះសម្បែង/រើទីលំនៅ		
2.11 លេខទូរសព្ទនាក់ជំងឺ			

3	ការស្វែងរកសេវាសុខភាព
3.1 តើអ្នកមានរោគសញ្ញាជំងឺរបេងណាមួយទេ មុនពេលធ្វើរោគវិនិច្ឆ័យ? (ប្រសិនបើគ្មានសូមបន្តទៅសំណួរ 3.3)	

លេខកូដអ្នកចូលរួម:_______________

☐ មាន ☐ អត់មាន

3.2 តើរយៈពេលយូរប៉ុណ្ណាដែលអ្នកមានរោគសញ្ញានោះ នៅមុនពេលចាប់ផ្តើមព្យាបាលជំងឺរបេង?

					**ពេលចាប់មានរោគសញ្ញា**	**(ថ្ងៃ ខែ ឆ្នាំ)**
ក្អក	☐ មាន	☐ គ្មាន	______	ថ្ងៃ	ថ្ងៃចាប់ផ្តើមមានរោគសញ្ញា	______
ក្អកធ្លាក់ឈាម	☐ មាន	☐ គ្មាន	______	ថ្ងៃ	ថ្ងៃចាប់ផ្តើមមានរោគសញ្ញា	______
មានកំហាក	☐ មាន	☐ គ្មាន	______	ថ្ងៃ	ថ្ងៃចាប់ផ្តើមមានរោគសញ្ញា	______
បែកញើសពេលយប់	☐ មាន	☐ គ្មាន	______	ថ្ងៃ	ថ្ងៃចាប់ផ្តើមមានរោគសញ្ញា	______
ស្រកទម្ងន់	☐ មាន	☐ គ្មាន	______	ថ្ងៃ	ថ្ងៃចាប់ផ្តើមមានរោគសញ្ញា	______
ឈឺទ្រូង	☐ មាន	☐ គ្មាន	______	ថ្ងៃ	ថ្ងៃចាប់ផ្តើមមានរោគសញ្ញា	______
អស់កម្លាំង	☐ មាន	☐ គ្មាន	______	ថ្ងៃ	ថ្ងៃចាប់ផ្តើមមានរោគសញ្ញា	______
គ្រុនក្តៅ	☐ មាន	☐ គ្មាន	______	ថ្ងៃ	ថ្ងៃចាប់ផ្តើមមានរោគសញ្ញា	______
ផ្សេងទៀត ៖ ______ (បញ្ជាក់)	☐ មាន	☐ គ្មាន	______	ថ្ងៃ	ថ្ងៃចាប់ផ្តើមមានរោគសញ្ញា	______

3.3 តើអ្នកបានស្វែងរកការព្យាបាល ឬ ដំបូន្មានអំពីរោគសញ្ញាទាំងនេះ នៅតាមកន្លែងដូចខាងក្រោមដែរឬទេ?

(គូសទាំងអស់ប្រសិនបើមាន)

តើកន្លែងណាដែលអ្នកទៅដំបូងគេ? (គូសរង្វង់នៅកន្លែងដំបូងគេ ដែលអ្នកទៅរកការព្យាបាល)

ពេទ្យរដ្ឋ	☐ បាទ/ចាស	☐ ទេ	មណ្ឌលសុខភាព	☐ បាទ/ចាស	☐ ទេ
ពេទ្យឯកជន/គ្លីនិក	☐ បាទ/ចាស	☐ ទេ	ឱសថស្ថាន,កន្លែងលក់ថ្នាំ	☐ បាទ/ចាស	☐ ទេ
ពេទ្យបូរាណ	☐ បាទ/ចាស	☐ ទេ	ផ្សេងទៀត (បញ្ជាក់)៖ ______	☐ បាទ/ចាស	☐ ទេ

3.4 តើអាគារសុខាភិបាលដែរនៅក្បែរអ្នកបំផុតមានចម្ងាយប៉ុន្មាន?

ក ពេលធ្វើរោគវិនិច្ឆ័យ&ព្យាបាល TB	______	ដើរ (នាទី)	______	ជិះ(នាទី)	______	ចម្ងាយ (គ.ម)
ខ សម្រាប់តែពេលព្យាបាល TB	______	ដើរ (នាទី)	______	ជិះ(នាទី)	______	ចម្ងាយ (គ.ម)

ផ្នែកទី ៤៖ សូមបំពេញលើតារាងដោយឡែក និងផ្តល់ព័ត៌មានអំពីការចំណាយមុន និង ពេលធ្វើរោគវិនិច្ឆ័យ?

5	**ការចំណាយលើការព្យាបាល**
	**ការចំណាយពាក់ព័ន្ធនឹង DOT**

5.1 តើអ្នកទៅយកថ្នាំ TB នៅកន្លែងណា?

☐ អាគារសុខាភិបាល/មន្ទីរពេទ្យ ☐ ផ្ទះ ☐ សហគមន៍ ☐ កន្លែងធ្វើការ ☐ ឱសថស្ថាន

5.2 ក. តើអ្នកត្រូវទៅទីនោះដើម្បីយកថ្នាំប៉ុន្មានដងក្នុងមួយសប្តាហ៍? ______ ដង/សប្តាហ៍

ខ. តើកំឡុងពេលប៉ុន្មានសប្តាហ៍ដែលអ្នកទៅទីនោះ? ______ សប្តាហ៍

5.3 តើអ្នកត្រូវចំណាយពេលប៉ុន្មានដើម្បីទៅកាន់ទីនោះ? (តែទៅប៉ុណ្ណោះ)

______ ដើរ (នាទី) ______ ជិះ (នាទី) ផ្សេងទៀត (បញ្ជាក់)៖ ______

លេខកូដអ្នកចូលរួម:________________

5.4 តើត្រូវចំណាយពេលជាមធ្យមប៉ុន្មានដើម្បីធ្វើដំណើរទៅកាន់ទីនោះដោយគិតទាំងរយៈពេលធ្វើដំណើរ និង រង់ចាំ?
____________ នាទី

5.5 ពីផ្ទះរបស់អ្នកទៅកាន់កន្លែង DOT (ដូចមានក្នុង 5.1), តើអ្នកត្រូវចំណាយប្រាក់ប៉ុន្មានប្រសិនបើអ្នកជិះ? (ទាំងទៅនិងមក)
____________ រៀល

5.6 តើត្រូវចំណាយប្រាក់ប៉ុន្មានសម្រាប់ការហូបចុកពេលធ្វើដំណើរតាមផ្លូវ ការរង់ចាំ & អាហារថ្ងៃត្រង់?
____________ រៀល

ការចំណាយពាក់ព័ន្ធនឹងការ ☐ទៅយកថ្នាំរបេង & ☐ដឹកជញ្ជូនថ្នាំរបេង

5.7 ក. តើអ្នកធ្វើដំណើរទៅអាគារសុខាភិបាល/មន្ទីរពេទ្យដើម្បីយកថ្នាំរបេងញឹកញាប់ប៉ុណ្ណា? ____________ ដង/ខែ

ខ. តើកំឡុងពេលប៉ុន្មានខែដែលអ្នកទៅយកថ្នាំរបេង? ____________ ខែ

5.8 តើត្រូវចំណាយពេលយូរប៉ុណ្ណាសម្រាប់ការធ្វើដំណើរទៅកាន់ទីនោះ? (តែទៅប៉ុណ្ណោះ)
________ ដើរ (នាទី) ____________ ជិះ (នាទី) ផ្សេងទៀត (បញ្ជាក់)៖ __________

5.9 តើការទៅកាន់ទីកន្លែងនោះ ត្រូវចំណាយពេលជាមធ្យមប៉ុន្មាន គិតទាំងការធ្វើដំណើរ និងការរង់ចាំ?
____________ នាទី

5.10 ពីផ្ទះរបស់អ្នកទៅកាន់អាគារសុខាភិបាល តើត្រូវចំណាយប្រាក់ប៉ុន្មានប្រសិនបើអ្នកជិះ? (ទាំងទៅនិងមក)
____________ រៀល

5.11 ប្រសិនបើអ្នកទៅអាគារសុខាភិបាលដើម្បីយកថ្នាំរបេង តើអ្នកត្រូវចំណាយប្រាក់ប៉ុន្មានសម្រាប់ការហូបចុកពេលធ្វើដំណើរតាមផ្លូវ ការរង់ចាំ & អាហារថ្ងៃត្រង់ ?
____________ រៀល

5.12 តើអ្នកត្រូវបង់ថ្លៃរដ្ឋបាល ឬ ថ្លៃដឹកជញ្ជូន ឬទេ នៅពេលអ្នកយកថ្នាំរបេង ? ☐ បាទ/ចាស ☐ ទេ

ក ប្រសិនបើបង់ ប៉ុន្មាន? ____________ រៀល

5.13 តើអ្នកត្រូវចំណាយលើការស្នាក់នៅទេ នៅពេលយកថ្នាំរបេង? ☐ បាទ/ចាស ☐ ទេ

ក ប្រសិនបើចំណាយ ប៉ុន្មាន? ____________ រៀល

5.14 តើអ្នកបានចំណាយប្រាក់ដើម្បីទិញថ្នាំរបេងដែរឬទេ? ☐ បាទ/ចាស ☐ ទេ

ក ប្រសិនបើបានបង់ ប៉ុន្មាន? ____________ រៀល

ការចំណាយពាក់ព័ន្ធនឹងការធ្វើតេស្តតាមដាន

5.15 តើអ្នកធ្លាប់បានទៅអាគារសុខាភិបាលដែរឬទេបន្ថែមលើការណាត់ជួបជាទៀងទាត់ដើម្បីធ្វើតេស្តតាមដានដោយចាប់តាំងពីពេលចាប់ផ្តើមការព្យាបាល? (ប្រសិនបើអត់ទេ សូមបន្តទៅសំណួរ 6.1) ☐ បាទ/ចាស ☐ ទេ

ក ប្រសិនបើធ្លាប់ ប៉ុន្មានដង? ________ ដង

5.16 ប្រសិនបើធ្លាប់ តើត្រូវចំណាយប្រាក់បន្ថែមទៀតដែរឬទេ នៅពេលដែលអ្នកទៅកាន់ទីនោះ? ☐ បាទ/ចាស ☐ ទេ

ក ប្រសិនបើដូច្នេះមែន តើត្រូវចំណាយលើអ្វី ហើយចំណាយប៉ុន្មានសម្រាប់ការទៅម្តងមួយលើក?

តំលៃចុះឈ្មោះ ________ តេស្តកំហាក ________ X-ray: ________
ផ្សេងទៀត ________ សរុប៖ ________

5.17 ចំពោះការតាមដាននេះ តើត្រូវចំណាយពេលជាមធ្យមប៉ុន្មាន គិតទាំងពេលធ្វើដំណើរ ការរង់ចាំ និង ការធ្វើតេស្ត? (ទាំងទៅ និង មក) ________ នាទី

5.18 តើជាមធ្យមអ្នកត្រូវចំណាយប្រាក់ប៉ុន្មាន សម្រាប់ការធ្វើដំណើរ ហូបចុក ការស្នាក់នៅ ពេលដែលទៅមួយលើកៗ?
ការធ្វើដំណើរ ________ ចំណីអាហារ ________ ការស្នាក់នៅ ________

6 ការចំណាយសម្រាប់អ្នករួមដំណើរ

6.1 បើមានសមាជិកគ្រួសារ/មិត្តភក្តិ/អ្នកជំនួយផ្នែក DOT ណាម្នាក់ ជូនអ្នកទៅកន្លែងព្យាបាលរបេង/ការធ្វើតេស្តតាមដាន /ការទៅបើកយកថ្នាំរបេង/និងទៅកន្លែងDOT តើអ្នកត្រូវចំណាយលុយបន្ថែមដែរឬទេ?
(ប្រសិនបើគ្មាន សូមបន្តទៅសំណួរ 7.1) ☐ បាទ/ចាស ☐ ទេ

a ប្រសិនបើមាន តើសមាជិកគ្រួសារ / មិត្តភក្តិ / អ្នកជំនួយផ្នែក DOT បានជូនអ្នកទៅ ប៉ុន្មានលើក?
ការព្យាបាល/ការទៅដើម្បីតាមដាន ________ ដង
ការចំណាយក្នុងការព្យាបាលមួយលើក ការធ្វើដំណើរ៖ ________ អាហារ៖ ________
ការស្នាក់នៅ៖ ________ ចំណាយព្យាបាលសរុប៖ ________ រៀល

6.2 តើសមាជិកគ្រួសារ/មិត្តភក្តិ/អ្នកជំនួយផ្នែក DOT រកបានប៉ុន្មានក្នុងមួយថ្ងៃ?
________ រៀល ☐ គ្មានចំណូល

6.3 ហេតុអ្វីបានជាមានអ្នកទៅជាមួយអ្នក? (គូសទាំងអស់ប្រសិនបើមាន)
☐ ផ្លូវឆ្ងាយ ☐ សុវត្ថិភាព ☐ ឧបសគ្គផ្នែករដ្ឋបាល ☐ ឈឺខ្លាំងពេកទៅម្នាក់ឯងមិនរួច
☐ ការព្យាបាលតម្រូវដូច្នេះ ☐ អ្នកជំងឺជាអនីតិជន ☐ ផ្សេងទៀត (បញ្ជាក់)៖ ________

7 ការចំណាយសម្រាប់ការសម្រាកពេទ្យ

7.1 តើអ្នកបានសម្រាកពេទ្យទេ ក្នុងពេលព្យាបាលជំងឺរបេង? (ប្រសិនបើអត់ សូមបន្តសំណួរ 8.1) ☐ បាទ/ចាស ☐ ទេ

ក ប្រសិនបើបានសម្រាកពេទ្យ តើអ្នកសម្រាកនៅពេទ្យប៉ុន្មានថ្ងៃ? ________ ថ្ងៃ

7.2 តើអ្នកត្រូវចំណាយប៉ុន្មាន ក្នុងការសម្រាក់ពេទ្យមួយថ្ងៃ?
ថ្លៃគ្រែ ________
អាហារ (មិនមែនផ្តល់ដោយមន្ទីរពេទ្យ)៖ ________ ថ្លៃធ្វើដំណើរ (ទៅមក) ________
ថ្នាំ* ________ តេស្ត** ________ ផ្សេងទៀត (បញ្ជាក់)៖ ________

* កុំបន្ថែមលើថ្លៃថ្នាំរបេងដូចមានក្នុងសំណួរ 5.14

** កុំបន្ថែមលើថ្លៃធ្វើតេស្តដែរ មានក្នុងសំណួរ 5.16 សរុប៖ ________ រៀល + តំលៃចុះឈ្មោះ ________

លេខកូដអ្នកចូលរួម:________________

7.3 តើមានសមាជិកគ្រួសារ//មិត្តភក្តិណាម្នាក់បាន**ស្នាក់នៅ**ពេទ្យជាមួយអ្នកទេ? ☐ បាទ/ចាស ☐ ទេ

ក ប្រសិនបើបានស្នាក់នៅ តើគេស្នាក់នៅជាមួយអ្នកបានប៉ុន្មានថ្ងៃ (គេងនៅទីនោះ)? ______ ថ្ងៃ

7.4 តើអ្នកត្រូវចំណាយប្រាក់បន្ថែមទេ នៅពេលសមាជិកគ្រួសារ/មិត្តភក្តិទៅកំដរអ្នកនៅពេទ្យ? ☐ បាទ/ចាស ☐ ទេ

ការស្នាក់នៅ (មន្ទីរពេទ្យ ឬកន្លែងផ្សេង)៖ ____________ អាហារ៖ ____________

ការធ្វើដំណើរ៖ ____________ ផ្សេងទៀត (បញ្ជាក់)៖ ____________ សរុប៖ ____________

7.5 តើសមាជិកគ្រួសារ/មិត្តភក្តិរកប្រាក់បានប៉ុន្មានក្នុងមួយថ្ងៃ?

____________ រៀល ☐ គ្មានចំណូល

7.6 តើមានសមាជិកគ្រួសារ/មិត្តភក្តិណាផ្សេងទៀតទៅសួរសុខទុក្ខអ្នក នៅមន្ទីរពេទ្យឬដែរទេ? ☐ មាន ☐ អត់

(ប្រសិនបើគ្មានសូមបន្តទៅសំណួរ 8.1)

ក ប្រសិនបើមាន តើសមាជិកគ្រួសារ/មិត្តភក្តិអ្នក ទៅសួរសុខទុកអ្នក ប៉ុន្មានដង? ______ ដង

ខ តើសមាជិកគ្រួសារ/មិត្តភក្តិណាផ្សេងទៀត ត្រូវចំនាយលុយប៉ុន្មានក្នុងការទៅសួរសុខទុក្ខអ្នកម្តងៗ ?

ការស្នាក់នៅ (មន្ទីរពេទ្យ ឬកន្លែងផ្សេង)៖ ____________ អាហារ៖ ____________

ការធ្វើដំណើរ៖ ____________ ផ្សេងទៀត (បញ្ជាក់)៖ ____________ សរុប៖ ____________ រៀល

គ តើការសួរសុខទុក្ខ មានរយៈពេលប៉ុន្មាន គិតទាំងពេលធ្វើដំណើរ? ______ នាទី

8	ការចំណាយផ្សេងទៀតសម្រាប់អាហារបន្ថែម

8.1 ដោយសារមានជំងឺរបេង តើអ្នកបានទិញអាហារបំប៉នបន្ថែមដែរឬទេ ដូចជា វីតាមីន សាច់ ភេសជ្ជៈបង្កើនថាមពល ភេសជ្ជៈគ្មានជាតិអាល់កុល ផ្លែឈើ និងថ្នាំផ្សេងៗ (ប្រសិនបើគ្មាន សូមបន្តទៅសំណួរ 9.1) ☐ បាទ/ចាស ☐ ទេ

ក ប្រសិនបើមាន តើមុខទំនិញណាមួយ? (បញ្ជាក់) (គូសទាំងអស់ ប្រសិនបើមាន)

☐ ផ្លែឈើ ☐ ភេសជ្ជៈ ☐ វីតាមីន/ថ្នាំបូរាណ ☐ សាច់ ☐ ផ្សេងទៀត (បញ្ជាក់) ______

8.2 ជាមធ្យម តើអ្នកត្រូវចំណាយប៉ុន្មានលើមុខទំនិញទាំងនេះ ក្នុងមួយខែ? ____________ រៀល

9	ជំងឺផ្សេងទៀត

9.1 តើអ្នកមានជំងឺរ៉ាំរ៉ៃណាមួយដែលទទួលការព្យាបាលទេ? (ប្រសិនបើគ្មាន សូមបន្តទៅសំណួរ 9.3)

☐ មុនពេលធ្វើរោគវិនិច្ឆ័យTB ☐ ក្រោយពេលធ្វើរោគវិនិច្ឆ័យTB ☐ មុន&ក្រោយធ្វើរោគវិនិច្ឆ័យTB ☐ អត់

បើមាន ជំងឺអ្វី? ក ____________ ខ ____________ គ ____________

9.2 តើអ្នកត្រូវចំណាយអ្វី បន្ថែមសម្រាប់ជំងឺទាំងនេះដែរឬទេ? ☐ បាទ/ចាស ☐ ទេ

ក ប្រសិនបើត្រូវចំណាយ តើត្រូវចំណាយប៉ុន្មានជាមធ្យម ក្នុងមួយខែ?

តេស្ត ____________ ថ្នាំ ____________ អាហារ ____________

	ការធ្វើដំណើរ ____________ ផ្សេងទៀត ____________________ សរុប៖ ______________	រៀល
9.3	តើជាមធ្យមអ្នកត្រូវចំណាយលើការថែទាំសុខភាពប៉ុន្មានក្នុងមួយខែ **មុនពេលមានជំងឺរបេង**? ______________	រៀល
9.4	តើ**បច្ចុប្បន្ន** អ្នកត្រូវចំណាយលើការថែទាំសុខភាពជាមធ្យមប៉ុន្មានក្នុងមួយខែ ? ____________________	រៀល

10	ប័ណ្ណបើកប្រាក់សង/ប័ណ្ណសមធម៌
10.1	តើអ្នកមានកម្មវិធីធានារ៉ាប់រងសុខភាព/ផ្នែកវេជ្ជសាស្រ្តឯកជន ឬរដ្ឋដែរឬទេ?<br>(ប្រសិនបើគ្មាន សូមបន្តទៅសំណួរ 10.2) ☐ មាន ☐ គ្មាន
ក	បើមាន ប្រភេទណា?<br>☐ ប្រាក់សំណង ☐ ប្រាក់ឧបត្ថម្ភផ្នែកវេជ្ជសាស្ត្រប្រចាំខែ ☐ ប័ណ្ណសមធម៌ ("ប័ណ្ណសម្រាប់អ្នកក្រ")<br>☐ មូលនិធិគ្រួសារ/សហគមន៍ ☐ គម្រោងរបស់ប៉ែកខាងលិច (កិច្ចសន្យា) ☐ ម្ចាស់ជំនួយ<br>☐ ផ្សេងទៀត (បញ្ជាក់)៖ ____________________
10.2	តើអ្នកបានដឹងអំពីប្រព័ន្ធប័ណ្ណសមធម៌នៅកម្ពុជាដែរទេ? ☐ ដឹង ☐ អត់ដឹង
10.3	តើអ្នកបានទទួលការបើកប្រាក់សងវិញសម្រាប់ការចំណាយពាក់ព័ន្ធនឹងការព្យាបាលTBដែរឬទេ?* ☐ បាន ☐ អត់ទេ<br>(ប្រសិនបើបើកប្រាក់សងវិញសម្រាប់មុនពេលធ្វើរោគវិនិច្ឆ័យ/ពេលធ្វើរោគវិនិច្ឆ័យ សូមប្រើផ្នែកទី 4)
ក	តើអ្នកបានទទួលសំណងវិញប៉ុន្មាន? ____________________ រៀល

11	ការចំណាយសម្រាប់ការដោះទ័ល
11.1	តើអ្នកបានខ្ចីប្រាក់ពីគេដែរឬទេទាំងមុន/ក្រោយរោគវិនិច្ឆ័យTB? (ប្រសិនបើអត់ទេ បន្តទៅសំណួរ 11.2)<br>☐ បានខ្ចី ☐ អត់ខ្ចី
ក	ប្រសិនបើបានខ្ចី តើខ្ចីគេប៉ុន្មាន? ________________ រៀល
ខ	តើអ្នកខ្ចីពីនរណា? ☐ គ្រួសារ ☐ អ្នកជិតខាង/មិត្តភក្តិ ☐ ធនាគារឯកជន<br>(គូសទាំងអស់ប្រសិនបើមាន) ☐ សហគមន៍ ☐ ផ្សេងទៀត (បញ្ជាក់)៖ ________________
គ	តើកម្ចីនោះមានការប្រាក់ប៉ុន្មាន? (%)<br>______________ % ☐ ពុំមានការប្រាក់ ☐ ខ្ញុំពុំតម្រូវឱ្យសងវិញទេ
11.2	តើអ្នកបានលក់ទ្រព្យសម្បត្តិសម្រាប់ការចំណាយលើជំងឺរបេងដែរឬទេ? ☐ បាទ/ចាស ☐ ទេ<br>ប្រសិនបើលក់ តើលក់អ្វី?
ក	☐ ដី ☐ បសុសត្វ ☐ មធ្យោបាយធ្វើដំណើរ/យានជំនិះ ☐ របស់របរក្នុងផ្ទះ<br>☐ ផលិតផលកសិកម្ម ☐ ផ្សេងទៀត (បញ្ជាក់)៖ ____________
ខ	តើទ្រព្យសម្បត្តិដែលអ្នកលក់មានតម្លៃប្រហាក់ប្រហែលប៉ុន្មាននៅលើទីផ្សារ? ____________________ រៀល
គ	តើអ្នកលក់វត្ថុនោះបានថ្លៃប៉ុន្មាន? ____________________ រៀល

លេខកូដអ្នកចូលរួម:________________

12	ព័ត៌មានសង្គមសេដ្ឋកិច្ច ស្ថានភាព និងប្រាក់ចំណូលរបស់បុគ្គល

12.1 តើនរណាជាអ្នករកចំណូលសំខាន់បំផុតនៅក្នុងគ្រួសារ?

☐ អ្នកជំងឺ ☐ ប្រពន្ធ/ម្តាយ ☐ ប្តី/ឪពុក ☐ គ្រួសារសាច់ញាតិច្រើន

☐ កូនប្រុស/កូនស្រី ☐ ផ្សេងទៀត (បញ្ជាក់)៖ ______________

12.2 តើការអប់រំខ្ពស់បំផុតកម្រិតណា?

ក អ្នកជំងឺ

☐ មិនចេះអក្សរ ☐ បឋមសិក្សា ☐ មធ្យមសិក្សា ☐ បញ្ចប់ការសិក្សា/សញ្ញាបត្រ ☐ ផ្សេងៗ(បញ្ជាក់) _____

ខ អ្នករកចំណូលសំខាន់បំផុត?

☐ មិនចេះអក្សរ ☐ បឋមសិក្សា ☐ មធ្យមសិក្សា ☐ បញ្ចប់ការសិក្សា/សញ្ញាបត្រ ☐ ផ្សេងៗ(បញ្ជាក់) _____

គ មេគ្រួសារ

☐ មិនចេះអក្សរ ☐ បឋមសិក្សា ☐ មធ្យមសិក្សា ☐ បញ្ចប់ការសិក្សា/សញ្ញាបត្រ ☐ ផ្សេងៗ(បញ្ជាក់) _____

12.3 តើអ្នកធ្វើការជាធម្មតាដែរឬទេ **មុនពេល**ធ្វើរោគវិនិច្ឆ័យTB?

☐ មានការងារធ្វើជាធម្មតា ☐ ឈប់សម្រាកដោយមានជំងឺ ☐ នៅរៀន ☐ ការងារផ្ទះ

☐ ទេ ធ្វើការងារម្តងម្កាល ☐ ចូលនិវត្តន៍ ☐ ផ្សេងៗទៀត (បញ្ជាក់) ______________

12.4 តើអ្នកធ្វើការជាធម្មតាដែរឬទេ ក្នុង**អំឡុងពេល**ព្យាបាលTB?

☐ មានការងារធ្វើជាធម្មតា ☐ ឈប់សម្រាកដោយមានជំងឺ ☐ នៅរៀន ☐ ការងារផ្ទះ

☐ ទេ ធ្វើការងារម្តងម្កាល ☐ ចូលនិវត្តន៍ ☐ ផ្សេងៗទៀត (បញ្ជាក់) ______________

ក តើមូលហេតុដែលមិនធ្វើការ គឺមកពីជំងឺរបេងមែនទេ? ☐ បាទ/ចាស ☐ ទេ

ខ ប្រសិនបើដូច្នេះមែន តើពេលណាជាពេលធ្វើការចុងក្រោយរបស់អ្នក? កាលបរិច្ឆេទ (ខែ ឆ្នាំ) ______________

12.5 តើ**បច្ចុប្បន្ន** អ្នកធ្វើការងារបានជាធម្មតាដែរឬទេ?

☐ មានការងារធ្វើជាធម្មតា ☐ ឈប់សម្រាកដោយមានជំងឺ ☐ នៅរៀន ☐ ការងារផ្ទះ

☐ ទេ ធ្វើការងារម្តងម្កាល ☐ ចូលនិវត្តន៍ ☐ ផ្សេងៗទៀត (បញ្ជាក់) ______________

ក តើមូលហេតុដែលមិនធ្វើការ គឺមកពីជំងឺរបេងមែនទេ? ☐ បាទ/ចាស ☐ ទេ

ខ ប្រសិនបើដូច្នេះមែន តើពេលណាជាពេលធ្វើការចុងក្រោយរបស់អ្នក? កាលបរិច្ឆេទ (ខែ ឆ្នាំ) ______________

12.6 តើប្រាក់ខែត្រូវបើកឱ្យអ្នកតាមរបៀបណា?

☐ សាច់ប្រាក់ ☐ សម្ភារ ☐ សាច់ប្រាក់ និងសម្ភារ ☐ ពុំមានប្រាក់ខែ

☐ ប្រាក់ខែផ្ទេរតាមធនាគារ ☐ ផ្សេងទៀត (បញ្ជាក់)៖ ______________

12.7 តើប្រាក់ចំណូលប្រចាំសប្តាហ៍របស់អ្នកប៉ុន្មានជាមធ្យម **មុនពេល**មានជំងឺរបេង?

(គិតទាំង ការទ្រទ្រង់ផ្នែកសុខមាលភាព ពិការភាព ឬ ការគាំទ្រផ្នែកសង្គមផ្សេងទៀត)

______________ រៀល/សប្តាហ៍ ☐ ពុំមានចំណូល

12.8 តើប្រាក់ចំណូលប្រចាំសប្តាហ៍របស់អ្នកប៉ុន្មានជាមធ្យម **ក្នុងពេល**មានជំងឺរបេង?

(គិតទាំង ការទ្រទ្រង់ផ្នែកសុខមាលភាព ពិការភាព ឬការគាំទ្រផ្នែកសង្គមផ្សេងទៀត)

________________ រៀល/សប្តាហ៍ ☐ ពុំមានចំណូល

ក ប្រសិនបើចម្លើយខុសពីសំណួរ 12.7 តើការប្រែប្រួលនោះមានពាក់ព័ន្ធនឹងជំងឺរបេងទេ? ☐ បាទ/ចាស ☐ ទេ

12.9 តើប្រាក់ចំណូលប្រចាំសប្តាហ៍របស់អ្នកប៉ុន្មានជាមធ្យម **ក្នុងពេលបច្ចុប្បន្ន?**

(គិតទាំង ការទ្រទ្រង់ផ្នែកសុខមាលភាព ពិការភាព ឬការគាំទ្រផ្នែកសង្គមផ្សេងទៀត)

________________ រៀល/សប្តាហ៍ ☐ ពុំមានចំណូល

ក ប្រសិនបើចម្លើយខុសពីសំណួរ 12.7 តើការប្រែប្រួលនោះមានពាក់ព័ន្ធនឹងជំងឺរបេងទេ? ☐ បាទ/ចាស ☐ ទេ

12.10 តើអ្នកធ្លាប់ឈប់ធ្វើការ/ឈប់ទៅសាលា/ឈប់ធ្វើការងារផ្ទះ ដោយសារតែជំងឺរបេងទេ? ☐ បាទ/ចាស ☐ ទេ

ក ប្រសិនបើឈប់ រយៈពេលប៉ុន្មាន? ____________________ ខែ

12.11 តើមានអ្នកណានៅផ្ទះ មើលថែអ្នក**ជាពិសេស**ទេ នៅមុន និង ក្រោយពេលធ្វើរោគវិនិច្ឆ័យជំងឺរបេង?

☐ បាទ/ចាស ☐ ទេ

ក ប្រសិនបើមាន រយៈពេលប៉ុន្មាន? _____________ សប្តាហ៍

ខ តើគាត់បោះបង់ ឬកាត់បន្ថយការងាររកចំណូលរបស់គាត់មកនៅផ្ទះ និងមើលថែទាំអ្នកដែរឬទេ? ☐ បាទ/ចាស ☐ ទេ

គ បើបាទ/ចាស តើគាត់បាត់បង់ប្រាក់ចំណូលជាមធ្យមប៉ុន្មានក្នុងមួយសប្តាហ៍? ______________________ រៀល

12.12 តើអ្នកធ្វើការងារញឹកញាប់កម្រិតណា មុនពេលអ្នកមានជំងឺរបេង?

☐ មួយឆ្នាំពេញ ☐ តាមរដូវ/ខែខ្លះនៃឆ្នាំ ☐ ការងារប្រចាំថ្ងៃ ☐ ផ្សេងៗ(បញ្ជាក់) _________

12.13 តើអ្នកត្រូវប្តូរការងារទេ នៅពេលមានជំងឺរបេង? ☐ បាទ/ចាស ☐ ទេ

12.14 តើការងារចម្បងរបស់អ្នកជាអ្វីនៅពេលបច្ចុប្បន្ន?

☐ លក់ដូរ/ផ្តល់សេវា ☐ កសិករ ☐ នៅផ្ទះ ☐ កម្មករផ្នែកផលិតកម្ម/សំណង់

☐ សិស្ស/និស្សិត ☐ គ្មានការងារ ☐ ការងាររដ្ឋ ☐ ផ្សេងទៀត (បញ្ជាក់)៖ ______________

12.15 តើជាមធ្យមអ្នកធ្វើការប៉ុន្មានម៉ោងក្នុងមួយថ្ងៃ?

ក **មុនពេល**មានជំងឺរបេង? __________ ម៉ោង

ខ **ក្នុងពេល**ព្យាបាល? __________ ម៉ោង

គ **ក្រោយពេល**ព្យាបាល? __________ ម៉ោង

12.16 ប្រសិនបើចម្លើយខុសគ្នា តើការប្រែប្រែនោះមានពាក់ព័ន្ធនឹងជំងឺរបេងដែរទេ? ☐ មាន ☐ ទេ

ក ប្រសិនបើចម្លើយខុសគ្នា តើមាននរណាម្នាក់ធ្វើការងារដែលអ្នកធ្លាប់ធ្វើពីមុនទេ? (គូសទាំងអស់ប្រសិនបើមាន)

☐ កូនស្រី ☐ កូនប្រុស ☐ ប្តី/ប្រពន្ធ ☐ មិត្តភ័ក្រ្ត ☐ អត់មាន ☐ សមាជិកគ្រួសារផ្សេងទៀត

12.17 តើអ្នកមានកូនគ្រប់អាយុចូលរៀន ឬ កូនដែលក្រោមអាយុចូលរៀនទេ? (ប្រសិនបើគ្មាន សូមបន្តទៅសំណួរ 12.19)

☐ មាន ☐ អត់មាន

12.18 កូនដល់អាយុចូលរៀនរបស់អ្នកបានទៅរៀនទៀងទាត់ទេ ក្នុងអំឡុងពេលអ្នកព្យាបាល?

☐ បានទៅ ☐ អត់បានទៅ

ក ប្រសិនបើមិនបានទៅ ហេតុអ្វី? (ជ្រើសចម្លើយតែមួយដែលចំបង)

☐ត្រូវជួយកិច្ចការផ្ទះ ☐អត់មានលុយសម្រាប់ថ្លៃសាលា ☐គាត់ឈឺដែរ

☐ត្រូវធ្វើការដើម្បីរកប្រាក់ ☐ផ្សេងទៀត (បញ្ជាក់)៖ ________________

ខ តើអ្នកមានកូនគ្រប់អាយុចូលរៀន ឬកូនដែលក្រោមអាយុចូលរៀនជួយធ្វើការរកប្រាក់សម្រាប់ចំណាយដោយសារជំងឺបេងដែរឬទេ? ☐មាន ☐អត់មាន

12.19 ប្រសិនបើអ្នកជ្រើសរើសអ្នកណាម្នាក់ឱ្យមកធ្វើការនៅក្នុងផ្ទះរបស់អ្នក តើអ្នកត្រូវចំណាយប្រាក់ជួលគេប៉ុន្មាន?

ក ពេលអ្នកឈឺ? ____________ រៀល/ថ្ងៃ

ខ ពេលអ្នកជា? ____________ រៀល/ថ្ងៃ

12.20 តើអ្នកអាចរកប្រាក់ចំណូលដោយខ្លួនឯងដោយមិនពឹងលើអ្នកដទៃដែរឬទេ? ☐បាទ/ចាស ☐ទេ

12.21 តើជំងឺរបេងបានជះឥទ្ធិពលដល់ជីវិតសង្គម ឬជីវិតឯកជនរបស់អ្នក តាមរបៀបណាមួយដែរឬទេ? (គូសទាំងអស់ប្រសិនបើមាន)

☐ទេ ☐លែងលះ ☐បាត់បង់ការងារ ☐ឈប់រៀន ☐កូនឈឺ

☐រស់នៅដាច់ពីប្តីប្រពន្ធ/ដៃគូ ☐រំខានដល់ជីវិតផ្លូវភេទ ☐ផ្សេងទៀត (បញ្ជាក់) ____________

12.22 តើជំងឺរបេងធ្វើឱ្យអ្នកមានបញ្ហាហិរញ្ញវត្ថុដែរឬទេ? ☐បាទ/ចាស ☐ទេ

13 ប្រាក់ចំណូល និងការចំណាយក្នុងគ្រួសារ

13.1 តើអាចប៉ាន់ស្មានបានទេថា ប្រាក់ចំណូលជាមធ្យមក្នុងគ្រួសារអ្នកក្នុងមួយខែប៉ុន្មាន**មុនពេល**អ្នកមានជំងឺរបេង? (រាប់បញ្ចូលមនុស្សទាំងអស់នៅក្នុងគ្រួសារគិតទាំង ប្រាក់ឧបត្ថម្ភពីឪពុកម្តាយ ប្រាក់ឧបត្ថម្ភសម្រាប់សុខភាពពីរដ្ឋ ឬជំនួយផ្នែកសង្គម) ប្រាក់ចំណូលអ្នកជំងឺ ____________ ប្រាក់សុខភាព ____________

ប្រាក់ចំណូលអ្នកផ្សេងទៀតក្នុងគ្រួសារ ____________ ជំនួយពីរដ្ឋាភិបាល ____________

ផ្សេងទៀត (បញ្ជាក់)៖ ____________ សរុប ____________ រៀល

13.2 តើអាចប៉ាន់ស្មានបានទេថា ប្រាក់ចំណូលជាមធ្យមក្នុងគ្រួសារអ្នកក្នុងមួយខែប៉ុន្មាន**ក្នុងពេល**អ្នកមានជំងឺរបេង?

ប្រាក់ចំណូលអ្នកជំងឺ ________ ប្រាក់ចំណូលអ្នកផ្សេងទៀតក្នុងគ្រួសារ ________ ប្រាក់សុខភាព ________

ជំនួយពីរដ្ឋាភិបាល ________ ផ្សេងទៀត (បញ្ជាក់)៖ ________ សរុប ________ រៀល

13.3 តើប្រាក់ចំណូលជាមធ្យមក្នុងគ្រួសាររបស់អ្នកក្នុងមួយខែទទួលបានប៉ុន្មាន**ក្រោយពេល**អ្នកមានជំងឺរបេង?

ប្រាក់ចំណូលអ្នកជំងឺ ____________ រៀល ប្រាក់ចំណូលផ្សេងទៀតក្នុងគ្រួសារ ____________ រៀល

ប្រាក់សុខភាព ____________ រៀល ជំនួយពីរដ្ឋាភិបាល ____________ រៀល

ផ្សេងទៀត (បញ្ជាក់)៖ ____________ រៀល សរុប ____________ រៀល

13.4 តើជាទូទៅមានមនុស្សប៉ុន្មាននាក់គេងនៅផ្ទះរបស់អ្នក? (គិតទាំងអ្នកជំងឺ) ____________ នាក់

13.5 តើមានសមាជិកគ្រួសារប៉ុន្មាននាក់ដែលមានការងារធ្វើ និងមានប្រាក់ខែ? (គិតទាំងអ្នកជំងឺនិងការទូទាត់ជាសម្ភារ

ឬផលិតផលកសិកម្ម) ________________ នាក់

13.6 ក្រៅពីរូបអ្នក តើមាននរណាម្នាក់ផ្សេងទៀតនៅក្នុងគ្រួសារអ្នក ទទួលការព្យាបាលជំងឺរបេងដែរឬទេ?

☐ បាទ/ចាស ☐ ទេ

ក បើមាន ប៉ុន្មាននាក់? ________________ នាក់

13.7 តើជាមធ្យម គ្រួសារអ្នកចំណាយលើចំណីអាហារប៉ុន្មានជារៀងរាល់ខែ នៅ**មុនពេល** **ក្នុងពេល** និង **ក្រោយពេល** អ្នកមានជំងឺរបេង (ប្រសិនបើផលិតផលដាំខ្លួនឯង) ប្រសិនបើចំណីអាហារដែលអ្នកបានទទួលទានក្នុងមួយខែ មុនពេល/ក្នុងពេល/ក្រោយពេលមានជំងឺរបេង ត្រូវបានយកទៅលក់នៅទីផ្សារ តើវាអាចមានតម្លៃប៉ុន្មាន? (បូកនឹងទឹកប្រាក់ជាមធ្យមដែលអ្នកត្រូវចំណាយសម្រាប់អាហារដែលមិនដាំខ្លួនឯង)

ក **មុនពេល** ________ រៀល

ខ **ក្នុងពេល** ________ រៀល ខ2. បើចម្លើយខុសពី ក តើបណ្តាលមកពីជំងឺរបេងមែនទេ? ☐ មែន ☐ មិនមែន

គ **ក្រោយពេល** ________ រៀល គ2. បើចម្លើយខុសពី ក តើបណ្តាលមកពីជំងឺរបេងមែនទេ? ☐ មែន ☐ មិនមែន

14	សូចនាករសង្គមសេដ្ឋកិច្ច

14.1 តើទឹកដែលអ្នកយកមកពិសាមានប្រភពចំបងមកពីណា?

☐ ទឹកភ្លៀង ☐ បឹង/ត្រពាំង/ទន្លេ ☐ អណ្តូងឯកជន/លូ

☐ ទឹកទុយោ ☐ ទឹកច្រកដប ☐ ទឹករដ្ឋ

14.3 តើបង្គន់អនាម័យប្រភេទណាដែលអ្នកប្រើប្រាស់?

☐ មិនមាន/តាមគុម្ពោតព្រៃ/វាលស្រែ ☐ បង្គន់មិនចាក់ទឹករួម ☐ បង្គន់មិនចាក់ទឹកផ្ទាល់ខ្លួន ☐ បង្គន់ចាក់ទឹក

14.4 តើផ្ទះរបស់អ្នកមានបន្ទប់ចំនួនប៉ុន្មាន?

☐ មួយ ☐ ពីរ ☐ បី ☐ បួន ឬ លើសពីនេះ

ក តើផ្ទះ/កន្លែងដែលអ្នកស្នាក់នៅជាកម្មសិទ្ធិរបស់អ្នកមែនទេ? ☐ មែន ☐ មិនមែន

14.5 តើអ្នកមាន? ចម្លើយអាចមានលើសពីមួយ

☐ វិទ្យុ ☐ ទូរទស្សន៍ ☐ ទូរស័ព្ទដៃ ☐ ទូរស័ព្ទលើតុ ☐ ទូរទឹកក

☐ ទូរដាក់ខោអាវ ☐ ម៉ាស៊ីនដេរ ☐ ម៉ាស៊ីនចាក់ CD/DVD ☐ ម៉ាស៊ីនភ្លើង

14.6 ប្រសិនបើរដ្ឋាភិបាលអាចផ្តល់សេវាមួយចំនួនដល់អ្នកដើម្បីជួយសម្រាលបន្ទុកជំងឺរបស់អ្នក និងគ្រួសារអ្នក តើអ្នកចង់បានសេវាអ្វី? សូមជ្រើសរើសតែមួយ

☐ សំបុត្របញ្ជាក់សម្រាប់ធ្វើដំណើរ ☐ សំបុត្របញ្ជាក់សម្រាប់អាហារ

☐ សេវាដែលមានប្រសិទ្ធភាពជាងនេះ ☐ ផ្សេងទៀត (បញ្ជាក់)៖ ________________

14.7 តើអ្នកអាចចំណាយប្រាក់បានប៉ុន្មានសម្រាប់ការព្យាបាលជំងឺរបេងនៅកន្លែងដំបូង?

☐ ក្រោម $20 ☐ $20-50 ☐ $51-100 ☐ ច្រើនជាង100$

លេខកូដអ្នកចូលរួម:_________________

មតិយោបល់របស់អ្នកសម្ភាសន៍
**ហត្ថលេខា**

តើអ្នកត្រូវចំណាយលុយម្តងៗចំនួនប៉ុន្មានក្នុងការទៅជួបពេទ្យ មុនពេលដឹងថាអ្នកមានជំងឺរបេង រួមបញ្ចូលទាំងការមកធ្វើរោគវិនិច្ឆ័យ?*

គ្រប់ចំនុចដែលមិនបានបំពេញ ត្រូវ ដាក់ N/A. ត្រូវបំពេញតែមួយបន្ទាត់ក្នុងការមកជួមម្តងៗ ហើយបំពេញអោយត្រូវតាមទៅលំដាប់លំដោយ

	A	B	C	D	E	F	G	H	I	J	K	L	M
ការណាត់ជួប	ប្រភេទនៃសេវាសុខាភិបាល**	កាលបរិច្ឆេទនៃការណាត់ជួប***	ចម្ងាយផ្លូវ(គីឡូម៉ែត)	រយៈពេលណាត់ជួបសរុប(គិតទាំងការធ្វើដំណើរទៅមកជានាទី)	តំលៃនៃការចុះឈ្មោះ/ការពិគ្រោះជំងឺ	តំលៃតេស្ត(ពិនិត្យកំហាក ឬ អ្វីផ្សេងទៀតលើកលែង x-ray)	តំលៃកាំរស្មីអ៊ិច(ថ្លៃបញ្ជូនទៅថត និងថ្លៃធ្វើដំណើរ)	តំលៃថ្នាំ(គ្រប់ប្រភេទ)	ថ្លៃធ្វើដំណើរ(ទាំងទៅទាំងមក)	ថ្លៃម្ហូបអាហារទាំងអស់	ថ្លៃស្នាក់នៅទាំងអស់	ចំណាយសរុបក្នុងការទៅម្តងៗ	ការធានារ៉ាប់រងឬសំណង
1				T:					Pt:	Pt:	Pt:	Pt:	
				O					G	G	G	G	
2				T:					Pt:	Pt:	Pt:	Pt:	
				O					G	G	G	G	
3				T:					Pt:	Pt:	Pt:	Pt:	
				O					G	G	G	G	
4				T:					Pt:	Pt:	Pt:	Pt:	
				O					G	G	G	G	
5				T:					Pt:	Pt:	Pt:	Pt:	
				O					G	G	G	G	
6				T:					Pt:	Pt:	Pt:	Pt:	
				O					G	G	G	G	

7				T:					Pt:	Pt:	Pt:	Pt:	
				O					G	G	G	G	
8				T:					Pt:	Pt:	Pt:	Pt:	
				O					G	G	G	G	
9				T:					Pt:	Pt:	Pt:	Pt:	
				O					G	G	G	G	
ចំណាយសរុបមុន និង ពេលធ្វើរោគវិនិច្ឆ័យ(ផលបូកនៃចំណាយសរុបម្តងៗ–សំណាងសរុបនៃធានារ៉ាប់រង)											រៀល		

* នេះរួមបញ្ចូលទាំងការធ្វើដំណើរទៅកាន់កន្លែងអ្នករៀបចំ ACF ផងដែរ (បើអ្នកជំងឺ ACF ជ្រើស យក"៦" សម្រាប់ "ប្រភេទនៃការផ្តល់សេវា")

**១.ពេទ្យរដ្ឋ ២.មណ្ឌលសុខភាព ៣.ពេទ្យឯកជន ៤.ឱសថស្ថាន កន្លែងលក់ថ្នាំ និង គ្រឿងទេស

៥. កន្លែងព្យាបាលដោយឱសថបុរាណ ៦. សហគមន៍ពិនិត្យជំងឺរបេង (ACF) ៧. ផ្សេងទៀត (បញ្ជាក់ក្នុងប្រអប់នេះ)

*** នេះគឺមានសារៈសំខាន់ខ្លាំងណាស់សម្រាប់ការសិក្សាស្រាវជ្រាវមួយនេះ។
